# Prevalence of tuberculosis in bovines in Pakistan during 2000–2024: a systematic review and meta-analysis

**DOI:** 10.3389/fvets.2025.1525399

**Published:** 2025-04-17

**Authors:** Siddique Sehrish, Xue-Tong Liu, Wen-Bo Lou, Shu-Ying Zhang, Emad Beshir Ata, Ge-Gui Yang, Qi Wang, Fan-Li Zeng, Xue Leng, Kun Shi, Riaz-Muhammad Azeem, Qing-Long Gong, Yu-Hao Song, Rui Du

**Affiliations:** ^1^College of Veterinary Medicine, Jilin Agricultural University, Changchun, Jilin, China; ^2^Ginseng and Antler Products Testing Center of the Ministry of Agricultural PRC, Jilin Agricultural University, Changchun, Jilin, China; ^3^College of Chinese Medicine Materials, Jilin Agricultural University, Changchun, Jilin, China; ^4^Department of Parasitology and Animal Diseases, Veterinary Research Institute, National Research Centre, Giza, Egypt; ^5^Department of Veterinary Medicine, College of Agriculture, Yanbian University, Yanji, China

**Keywords:** Pakistan, prevalence, raw milk, bovine tuberculosis, zoonosis

## Abstract

**Background:**

Bovine tuberculosis (bTB) primarily caused by *Mycobacterium bovis (M. bovis)*, is a globally prevalent zoonotic infectious disease of cattle and other livestock and wildlife species. Pakistan is the fourth-largest milk-producing country in the world, with approximately 212 million animals. Livestock farming provides a livelihood for almost 8 million families. Moreover, there is currently no effective control program and national data in place. Therefore, we constructed a first meta-analysis on the prevalence of bovine tuberculosis in Pakistan. This study aimed to provide an overview of bovine tuberculosis in this country and identify the risk factors associated with its prevalence.

**Methods:**

We searched Science Direct, Pubmed, Base, Green File-Poly U Library, Google Scholar, and additional articles were also identified manually from reference lists of articles generated in database search, systemically for papers that presented bTB prevalence data, published in English language between January 1, 2000, and April 30 2024. A total of 35 published articles were selected for inclusion in the meta-analysis.

**Results:**

The estimated overall prevalence of bTB was found to be 6.06% [95% CI: 4.67–7.87]. Cattle were more susceptible to infection, with a higher prevalence (6.44% [95% CI: 4.04–10.26]) compared to buffalo (5.54% [95% CI: 3.13–9.81]). The prevalence determined by PCR (5.65% [95% CI: 3.33–5.98]) was much similar to that of TST (5.61% [95% CI: 4.20–7.50]) with no significant difference. Milk samples showed the highest prevalence (14.66% [95% CI: 7.38–29.11]), particularly due to the consumption of unpasteurized milk, improper handling of dairy products and suckling by calves from the infected cows. Furthermore, the analysis considered effect of various potential risk factors (age, weight, breed, body condition score, herd size, animal status) along with different geographical factors (longitude, latitude, altitude, humidity, rainfall, temperature, climate) associated with bTB prevalence, which should be considered when developing future disease surveillance and control programs.

**Conclusion:**

In Pakistan bTB was widely distributed throughout the country, as a neglected zoonotic disease. Long-term disease prevalence monitoring should be recommended along with the need to improve diagnostic techniques, enhance farm management practices, and implement targeted surveillance to protect both animal and public health.

## 1 Introduction

The farm animals are playing a marked role in keeping the global food security (1–3). They were subjected to different pathogens that affect their productivity (4–7). Bovine tuberculosis (bTB) is a chronic, debilitating granulomatous disease caused by *Mycobacterium bovis* (*M. bovis*) and belongs to the *Mycobacterium tuberculosis* complex (MTC) (8). It is also a zoonotic disease, with major infections including human tuberculosis (9). The infected animals exhibit asymptomatic phase in the early stages of infection, then gradually develop emaciation, low-grade fluctuating fever, enlarged draining lymph nodes, and udder induration can be observed in the later stages of the disease (10). When the digestive tract is affected, the clinical symptoms vary from diarrhea and constipation to cough and dyspnea (11).

Bovine tuberculosis is widespread throughout the world, with the highest prevalence observed in Asian and African countries, except Antarctica (12). According to the World Organization for Animal Health (WOAH), between 2017 and 2018, 44% of countries reported the disease occurrence. Of these nations, the majority (62%) reported illnesses in livestock alone, although 35% reported infections in both livestock and wildlife (13). Globally, the disease is estimated to impact more than 50 million cattle (14). The largest prevalence of infected herds were reported in India, where 7.3% of farm and dairy cattle have bovine tuberculosis (15). The following countries are free bTB, based on current statistics: Norway, Austria, Switzerland, Luxembourg, Jamaica, Latvia, Slovakia, Iceland, Estonia, Canada, Lithuania, Finland, Barbados, Singapore, Australia, Sweden, the Czech Republic and Denmark. Several European countries, as well as the United States, New Zealand, and Japan, have programs in place to eradicate bovine tuberculosis this disease (16). The main etiological agent is *M. bovis* (Supplementary Table S4), but other *Mycobacterium* species, such as *M. tuberculosis*, *M. caprae* (17), *M. orygis* (18), *M. microti* (19), and *M. africanum* can also infect various livestock and wildlife (20, 21). Although the principal reservoir host of bTB include cattle, it is prevalent in other species-like human, pigs, goats, buffaloes, primates, dogs, deers, possums, badgers, bison and wild animals (22, 23), also posing threat to some endangered species (24).

The transmission risk of bovine tuberculosis is influenced by pathogen, host, and environmental factors (25). Depending on the site of infection in the body, bacteria can be found in vaginal secretions, respiratory secretions, milk, feces, semen, urine, and exudates from lesions (such as lymph node drainage and certain skin lesions) (26–28). The predominate route of transmission among bovines is inhalation. Susceptible animals breathe in infectious aerosol droplets released in the respiratory secretions of infected animals, particularly in overcrowded or poorly ventilated environments (29). Direct contact with infected skin wounds and mucous membranes is another common route of transmission (30). Ingestion can also occur when animals consume feed, water or surfaces contaminated with *M. bovis* from infected secretions or excretions (31). Vertical transmission though rare, can occur in utero or postpartum (32). Calves are highly susceptible to infection through contaminated milk or colostrum from infected dams. The disease is primarily transmitted from bovines to humans through unpasteurized dairy products and direct contact with infected animals or their bodily fluids such as during handling or slaughtering (33, 34). Additionally, *M. bovis* could be transmitted by consumption of infected raw or undercooked meat and other animal-derived tissues (35).

The bovine tuberculosis results in considerable economic losses globally, causing an estimated loss of USD 3 billion annually in the form of decreased production along with higher mortality rates, culling, movement and trade restrictions. European Union (EU) legislation mandated that disease eradication is important for both public health and free trade facilitation of livestock products globally (36, 37). Azami and Zinsstag attributed the economic costs of bTB to several factors, including a 10–18% decrease in milk production, a 10–25% loss in productive efficiency, higher rates of edible organ condemnation, 15% reduction in meat production, and increased mortality (38). Throughout the history, humans consumed cattle meat and milk as basic food sources so zoonotic transmission were higher. Studies have identified the same strain of *M. bovis*, responsible for bTB, can be found in both humans and animals. This suggests potential “spillover” mechanism from animals to humans (39). Many developed countries have controlled the bTB, under active national control programs. Although maintaining bTB-free status and total eradication remains difficult because of spill-over possibility from animal-reservoir hosts (40).

The prevalence of bovine tuberculosis is influenced by various potential risk factors including both animal and herd levels. Animal-specific factors include sex, age, breed, body condition, weight and mode of transmission (41–43). Herd-level factors include herd size, biosecurity measures and overall farm management practices (16, 44). Prevalence is greatly influenced by geographic and environmental factors. Climatic conditions such as temperature, humidity and seasonal variations also play important role (45). Understanding these factors is crucial for the development of successful disease management and prevention strategies.

Pakistan faces significant challenge with bovine tuberculosis control. In 1969, the district of Faisalabad reported the first case of bTB with (6.72%) prevalence in dairy animals (46). Currently, Pakistan ranks 5th globally in the incidence of new human TB cases, with over than 500,000 cases reported annually. Within the WHO Eastern Mediterranean Region, this burden accounts for total of (61%) of all TB recorded cases. Moreover, 63% of population resides in rural areas of Pakistan, and a significant portion (62%) of population is directly or indirectly involved with livestock activities (47). The overlap between human-livestock contacts and high TB prevalence poses a significant public health concern. According to the Pakistan economic survey during 2023–2024, Pakistan reveals substantial bovine population with an estimation of 57.5 million cattle and 46.3 million buffaloes. Despite this huge significant livestock sector, data on bTB prevalence is still incomplete. To address this gap, we conducted this first-ever national meta-analysis to determine the prevalence of bTB in Pakistan. Furthermore, various factors that might influence the occurrence of the disease were also investigated, including geographical location, sampling year and season, detection methods, and animal characteristics (age, sex, and weight). The quality of the original studies was also assessed. Geographical factors including longitude, latitude, altitude, rainfall, humidity, temperature and climate, were also analyzed to assess their association with bTB infection.

## 2 Materials and methods

### 2.1 Search strategy

A meta-analysis was conducted following the guidelines of PRISMA (2009) (Supplementary Tables S1, S4) (48). We searched the published research literature on bovine tuberculosis through four databases and one search engine: Science Direct, PubMed, Base, Green File-Poly U Library, Google Scholar and additional articles were also identified manually from the reference lists of articles generated in the database search. All the relevant published literature on bovine tuberculosis in Pakistan was retrieved during the period from 1 January 2000 to 1 April 2024. This search was carried out on 3rd June 2024 and Endnote (version X.20) was used to record the retrieved articles.

#### 2.1.1 Search terms

In Science Direct database, the keywords “Bovine,” “Cattle,” “Buffalo,” “Tuberculosis” and “Pakistan” were used for searching. In the base and Green File- Poly U Library, the used terms were “Cattle,” “Buffalo,” “Tuberculosis” and “Pakistan.” For Google Scholar, the words “Cattle,” “Bovine,” “Buffalo,” “*Mycobacterium bovis*,” “*M. bovis*,” “and bovine tuberculosis,” “Pakistan” were used.

The following formulas and MeSH terms “Cattle,” “Buffalo,” “Tuberculosis” and “Pakistan” were used in Pubmed. Boolean operators “AND” were used to connect MeSH terms and “OR” to connect the entry terms.

(“Cattle”[Mesh]) OR (Cow)) OR (Cows)) OR (*Bos indicus*)) OR (*Bos indicus* Cattle)) OR (*Bos indicus* Cattles)) OR (Cattle, *Bos indicus*)) OR (Cattles, *Bos indicus*)) OR (Indicine Cattle)) OR (Cattle, Indicine)) OR (Cattles, Indicine)) OR (Indicine Cattles)) OR (Zebu)) OR (Zebus)) OR (Holstein Cow)) OR (Cow, Holstein)) OR (Dairy Cow)) OR (Cow, Dairy)) OR (Dairy Cows)) OR (Beef Cow)) OR (Beef Cows)) OR (Cow, Beef)) OR (*Bos grunniens*)) OR (Yak)) OR (Yaks)) OR (*Bos taurus*)) OR (Taurine Cattle)) OR (Cattle, Taurine)) OR (Cattles, Taurine)) OR (Taurine Cattles)) OR (Taurus Cattle)) OR (Cattle, Taurus)) OR (Cattles, Taurus)) OR (Taurus Cattles)) OR (Cow, Domestic)) OR (Domestic Cow)) OR (Domestic Cows)).

AND (((((((“Buffaloes”[Mesh]) OR (Buffalo)) OR (Bubalus)) OR (Syncerus)) OR (Water Buffaloes)) OR (Water Buffalo)) OR (Buffalo, Water))).

AND ((((“Tuberculosis, Bovine”[Mesh]) OR (Bovine Tuberculoses)) OR (Bovine Tuberculosis)) OR (Tuberculoses, Bovine))).

AND ((“Pakistan” [Mesh]) OR (Islamic Republic of Pakistan)).

### 2.2 Selection criteria

Eligible studies were selected according to the following inclusion and exclusion criteria.

#### 2.2.1 Inclusion criteria

- Studies specified for bovine tuberculosis in Pakistan.
- Published between 2000 and 2024.
- Data must include both (total sample size and bTB prevalence).
- Description of clear detection methods.
- Adequate sample size, >30 animals.

#### 2.2.2 Exclusion criteria

- Studies investigating other than mentioned disease and conducted outside of Pakistan.
- Articles content that did not match with titles and abstracts.
- Hosts were not bovine.
- Full-text articles were not available.
- Data repetition in the articles.

### 2.3 Data extraction

Two reviewers XUL and WBL extracted data from qualified studies. The following information was reported in standardized forms using Microsoft Excel 2021: first author, publication year, number of samples (total and positive), study period, sample classification, detection method, characteristics of animal (age, gender, body condition score, weight, herd size), additional factors (animal status, lactation status, lactation length, parity), and geographical factors (longitude, latitude, altitude, rainfall, humidity, temperature, climate).

### 2.4 Quality assessment

The quality of eligible publications was evaluated according to criteria derived from Grading of Recommendations Assessment, Development and Evaluation Method (GRADE) (49). Each study received one point if it met one of the following criteria: (clear detection method, random sampling, sampling method, sampling time, four or more risk factors). Based on scoring system, studies were assigned to three quality categories (Table 1). High quality as 3–4 points, medium quality 2 points and low quality 1 points.

**Table 1:** The studies of tuberculosis in bovines in Pakistan.

References	Sampling time	Detection method*	No. tested	No. positive	Prevalence	Quality score
Northeast Pakistan						
Hamid et al. (2003) (123)	UN	SCITT	1,000	73	0.073	3
Khan et al. (2007) (124)	UN	CIDT	2,526	321	0.1272	2
Mumtaz al. (2008) (125)	UN	SITT	31	3	0.0967	2
Javed et al. (2009) (126)	2006.08–2007.02	CIDT	395	9	0.0227	3
Javed et al. (2010) (76)	2007.05–2007.07	SCCIT	1,092	28	0.0256	3
Arshad et al. (2012) (109)	2005–2008	SICTT	1,052	26	0.0247	4
Khan et al. (2012) (127)	UN	SCCIDTT	100	2	0.02	2
Tipu et al. (2012) (128)	2007.05–2008.02	SCIDTT	1,000	134	0.134	4
Ghumman et al. (2013) (78)	2006.07–2009.10	ITT	17,601	2084	0.1184	2
Javed et al. (2013) (129)	2007.05–2007.07	SCCIT	521	12	0.023	3
Ali et al. (2014) (75)	2010.07–2012.06	SICTT	1,031	28	0.0271	3
Mahmood et al. (2014) (130)	UN	SCCIT	107	8	0.0747	3
Akhtar et al. (2015) (72)	UN	SICCITT	215	53	0.246	3
Waqas et al. (2015) (131)	UN	ZN-Staining	400	5	0.0125	2
Aslam et al. (2019) (132)	UN	IDTT	265	28	0.1056	3
Rehman et al. (2021) (55)	UN	TST	627	27	0.043	2
Zahoor et al. (2021) (133)	UN	CCIT	340	14	0.041	2
Tariq et al. (2024) (89)	UN	SITT	192	18	0.09375	3
Northwest Pakistan						
Azam et al. (2014) (134)	UN	SICTT	200	4	0.02	3
Khan et al. (2014) (135)	2011.07–2011.11	Culture	302	27	0.0894	4
Basit et al. (2015) (136)	UN	PCR	107	5	0.0467	2
Noorahim et al. (2015) (137)	UN	TST	236	13	0.0551	3
Khattak et al. (2016) (138)	2014.06	CCIT	556	32	0.0575	4
Nawaz et al. (2017) (139)	UN	SSIDT	276	22	0.0797	2
Basit et al. (2018) (140)	UN	PCR	126	8	0.0634	3
Ullah et al. (2019) (141)	2016.01–2016.12	CCIT	2,400	141	0.0587	3
Southwest Pakistan						
Batool et al. (2017) (142)	UN	Tuberculin	217	3	0.0138	3
Southeast Pakistan						
Memon et al. (2019) (143)	2015.03–2016.09	Meat inspection & Necropsy	943	51	0.054	3
Qazi et al. (2012) (144)	UN	SITT	187	10	0.0534	1
Leghari et al. (2016) (145)	UN	Culture	160	4	0.025	3
Memon et al. (2017) (146)	UN	CIDT	1,000	144	0.144	3
Malhi et al. (2018) (147)	UN	Culture	120	18	0.15	3
Memon et al. (2018) (148)	UN	Lateral Flow Technique	800	47	0.0587	2
Leghari et al. (2020) (92)	UN	Culture	160	64	0.4	4
Mazari et al. (2022) (149)	UN	Culture	800	50	0.0625	3

SCITT*, Single Cervical Tuberculin Test; CIDT*, Comparative Intradermal Tuberculin Test; SITT*, Single Intradermal Comparative Tuberculin Test; SCCIT*, Single Cervical Comparative Intradermal Tuberculin Test; SICTT*, Single Intradermal Cervical Tuberculin Test; SCCIDTT*, Single Cervical Comparative Intradermal Tuberculin Test; SCIDTT*, Single Cervical Intradermal Tuberculin Test; ITT*, Intradermal Tuberculin Test; SICCITT*, Single Intradermal Comparative Cervical Tuberculin Test; ZN-Staining*, Ziehl-Neelsen Staining (microscopic examination for acid-fast bacilli); IDTT*, Intradermal Delayed-Type Hypersensitivity Test; TST*, Tuberculin Skin Test (similar to ITT); CCIT*, Cervical Comparative Intradermal Tuberculin Test; SSIDT*, Single Subcutaneous Intradermal Tuberculin Test; PCR*, Polymerase Chain Reaction (for detecting DNA/RNA of *M. bovis*); Culture *, Isolation and growth of *M. bovis* from clinical samples; Lateral Flow Technique*, Rapid diagnostic test using lateral flow immunoassay.

### 2.5 Statistical analysis

According to PRISMA statement, meta-analysis was performed and the “meta” package in R Studio (version 4.4.1) was used for the model’s estimation (45, 50). Before meta-analysis, four methods of data transformation were tested to bring the data closer to the normal distribution: original rate (PRAW), logarithmic conversion (PLN), logit transformation (PLOGIT), arsenic transformation (PAS) and double arsenic transformation (PFT) (Table 2). The conversion rate is based on the Shapiro–Wilk normality test. According to criteria, the *p-value > 0.05* and W-value closer to 1, PLN was finally chosen for exchange conversion rate (W = 0.978, *p* = 0.713). Forest plots were used to visualize the results of analysis and to determine heterogeneity between studies. Forest plot gives the results of both common effect model as well as random effects model. Due to strong heterogeneity of the included studies, random-effects model was chosen for meta-analysis. To determine the statistical difference of heterogeneity between the included studies, Cochran’s Q-test (51), the I^2^ statistic and χ^2^ test (*p < 0.05*) were used. To verify presence of publication bias, a funnel plot, and an Eager’s test were used. Sensitivity analysis was performed to check the reliability of meta-analysis results. The code for R in meta-analysis is shown in (Supplementary Table S2).

**Table 2:** The normality test for the original rate and various transformations of the rate.

Conversion form	*W*	*p*
PRAW	0.71886	<0.05
PLN	0.97862	>0.05
PLOGIT	0.97305	>0.05
PAS	0.88102	<0.05
PFT	0.87379	<0.05

“PRAW”: original rate; “PLN”: logarithmic conversion; “PLOGIT”: logit transformation; “PAS”: arcsine transformation; “PFT”: double-arcsine transformation.

In meta-analyses, heterogeneity is a crucial factor to consider when evaluating studies. To explore the potential sources of heterogeneity, research data were analyzed using subgroup analyses and univariate meta-regression analyses. These analyses identified factors that contributed to heterogeneity among studies. These potential sources consist of study period, detection methods, age, gender, specie, breed, weight, animal status (pregnant, non-pregnant, lactating, non-lactating, parity), Body condition score (BCS), survey area, sample classification, quality of articles (Table 3). To further analyze the potential sources of heterogeneity, we also conduct an analysis of geographical factor sub-groups, including longitude, latitude, altitude, rainfall, humidity and climate (Table 4). Our meta-analysis is not registered in the Cochrane database as it does not include review agreement. Due to the absence of significant differences in some of the sub-group analyses, a point estimate will be used to determine bTB prevalence in our study.

**Table 3:** The pooled prevalence of tuberculosis in bovines in Pakistan.

		No. studies	No. tested	No. positive	% (95% CI*)	Heterogeneity			Univariate meta-regression	
						χ^2^	*p*-value	I^2^ (%)	*p*-value	Coefficient (95% CI)
Region^a^										
	Northwest Pakistan (Khyber Pakhtunkhwa)	7	4,003	248	6.43% (5.48–7.53)	6.68	0.35	10.2%		
	Northeast Pakistan (Punjab)	18	28,495	2,873	5.54% (3.76–8.16)	384.56	<0.0001	95.6%		
	Southeast Pakistan (Sindh)	8	4,170	388	8.72% (4.87–15.58)	257.99	<0.0001	97.3%	>0.05	−0.304485 (−0.82–0.21)
	Southwest Pakistan (Balochistan)	1	217	3	–	–	–	–		
Study period										
	2005–2007	5	7,733	797	3.32% (1.87–5.89)	208.32	<0.01	97.1%	<0.05	0.70 (0.06–1.35)
	2007–2011	5	14,230	1,523	9.24% (6.49–13.16)	47.44	<0.01	89.5%		
	2011–2017	5	1,031	28	5.90% (3.54–9.84)	35.93	<0.01	88.9%		
Survey area										
	Abbatoirs	6	2,828	157	5.34% (3.38–8.45)	19.99	<0.01	75.0%		
	Farms	19	28,139	2,889	6.14% (4.15–9.09)	373.85	<0.01	95.2%		
	Rural/Urban/Towns	8	5,383	453	8.71% (4.91–15.44)	290.14	<0.01	97.6%	>0.05	0.39 (−0.25–1.03)
Sample classification										
	Milk	11	1,477	208	14.66% (7.38–29.11)	211.97	<0.01	95.3%	>0.05	0.22 (−0.82–1.28)
	Nasal swabs	8	1,526	130	11.62% (5.19–26.02)	127.81	<0.01	94.5%		
Detection methods^b^										
	Culture	5	1,047	61	4.67% (2.50–8.72)	15.49	<0.01	74.2%		
	TST	25	33,022	3,214	5.61% (4.20–7.50)	491.16	<0.01	95.1%	>0.05	0.15 (−0.47–0.78)
	PCR	2	233	13	5.65% (3.33–9.58)	0.31	0.58	0.0%		
Specie										
	Bovine	14	24,836	2,523	6.07% (4.11–8.97)	262.63	<0.01	95.1%		
	Cattle	12	5,306	415	6.44% (4.04–10.26)	312.11	<0.01	96.5%		
	Buffalo	9	6,943	578	5.54% (3.13–9.81)	195.19	<0.01	95.9%	>0.05	−0.11 (−0.71–0.49)
Gender										
	Male	17	3,037	131	4.41% (2.63–7.39)	223.41	<0.01	91.9%	>0.05	−0.40 (−1.05–0.25)
	Female	18	10,462	876	6.72% (4.47–10.08)	428.17	<0.01	95.6%		
Age										
	1–3	3	1859	64	3.50% (2.75–4.46)	1.94	0.58	0.0%	<0.05	−1.04 (−2.06 to −0.01)
	4–6	9	1,288	138	10.26% (6.31–16.69)	78.51	<0.01	89.8%		
	7–10	20	5,337	647	8.02% (5.09–12.64)	1238.46	<0.01	98.0%		
Weight										
	200–300	5	650	9	2.26% (0.82–6.23)	11.65	0.04	57.1%		
	300–400	9	3,568	446	3.56% (1.12–11.33)	5.20	0.01	61.5%		
	400–500	2	362	22	7.75% (4.20–14.28)	480.57	<0.01	97.1%	<0.05	1.07 (0.04–2.09)
Animal status										
	Pregnant	6	713	222	10.75% (2.93–39.50)	89.80	<0.01	94.4%	>0.05	−0.25 (−1.68–1.16)
	Non-Pregnant	11	1,450	317	8.84% (4.00–19.56)	253.48	<0.01	96.1%		
Lactation status										
	Lactating	10	3,886	289	9.93% (5.80–17.02)	271.40	<0.01	95.2%	>0.05	0.42 (−0.40–1.26)
	Non-Lactating	8	1,126	63	6.75% (3.90–11.67)	43.28	<0.01	83.8%		
Lactation length										
	0–3	4	881	12	1.97% (1.20–3.25)	4.67	0.46	0.0%	<0.05	−1.38 (−2.36 to −0.39)
	3–6	3	533	29	7.54% (2.80–20.30)	13.92	<0.01	85.6%		
	6–10	4	526	58	6.32% (2.57–15.54)	39.64	<0.01	87.4%		1.35 (0.23–2.47)
Parity										
	1–3	4	634	83	7.65% (2.31–25.40)	31.91	<0.01	90.6%		
	3–6	4	853	272	14.10% (4.31–46.12)	141.81	<0.01	96.5%	>0.05	0.76 (−0.90–2.44)
BCS										
	Good	6	1,630	34	2.43% (1.29–4.60)	13.93	0.02	64.1%	<0.05	−1.81 (−2.83 to −0.79)
	Poor	6	1809	280	13.51% (6.39–28.58)	305.56	<0.01	98.0%		
	Fair	2	176	46	17.66% (3.63–85.88)	13.06	<0.01	92.3%		
Herd size										
	1–50	7	3,331	209	7.09% (4.81–10.44)	166.18	<0.01	88.0%		
	51–100	4	1840	44	2.50% (1.88–3.33)	0.18	0.98	0.0%	<0.05	−1.27 (−2.20 to −0.33)
	101–500	4	558	67	10.59% (3.90–28.75)	39.57	<0.01	92.4%		
Breed										
	Local	13	7,241	518	5.33% (3.32–8.56)	316.55	<0.01	93.4%	<0.05	−0.81 (−1.60 to −0.01)
	Exotic	4	608	78	13.65% (5.44–34.24)	70.97	<0.01	95.8%		
	Cross-bred	6	1,008	159	9.72% (3.85–24.56)	71.69	<0.01	93.0%		
Quality level										
	1–2	11	22,995	2,540	5.74% (3.89–8.45)	115.75	<0.01	91.4%		
	3–4	24	14,090	976	6.24% (4.46–8.73)	632.02	<0.01	96.4%	>0.05	0.12 (−0.45–0.69)
Total		35	37,085	3,516	6.06% (4.67–7.87)	770.91	<0.01	96%		

CI*, confidence interval.

Region^a^: Northwest Pakistan: Khyber Pukhtunkhawa; Northeast Pakistan: Punjab; Southeast Pakistan: Sindh; Southwest Pakistan: Baluchistan.

Methods^b^: TST, Tuberculin Skin Test; PCR, Polymerase Chain Reaction (for detecting DNA/RNA of *Mycobacterium tuberculosis*); Culture, Isolation and growth of *Mycobacterium tuberculosis* from clinical samples.

**Table 4:** Geographical factors affecting the prevalence of bovine tuberculosis in Pakistan.

	No. studies		No. tested	No. positive	% (95% CI*)	Heterogeneity			Univariate meta-regression	
						χ^2^	*p*-value	I^2^ (%)	*p*-value	Coefficient (95% CI)
Latitude										
	24–28^o^	8	4,170	388	9.29% (5.56–15.53)	382.63	<0.01	97.1%	<0.05	0.58 (0.04–1.12)
	28–31^o^	2	557	11	5.04% (3.43–7.40)	396.58	<0.01	95.2%		
	31–35^o^	25	32,358	3,111	6.24% (5.27–7.39)	11.94	<0.01	41.4%		
Longitude										
	66–69^o^	9	4,387	391	8.21% (4.80–14.06)	400.53	<0.01	97.0%		
	69–72^o^	8	4,218	301	6.43% (5.48–7.53)	6.68	−0.35	10.2%		
	>72^o^	18	28,480	2,824	5.11% (3.50–7.44)	395.38	<0.01	95.2%	>0.05	−0.39 (−0.91–0.11)
Altitude										
	<100 m	4	3,620	389	8.66% (4.05–18.52)	208.58	<0.01	97.6%		
	100–1,500 m	26	14,410	951	5.71% (4.28–7.62)	598.88	<0.01	95.5%	>0.05	−0.31 (−0.87–0.25)
	>1,500 m	5	19,055	2,176	6.56% (2.79–15.40)	107.31	<0.01	95.3%		
Rainfall										
	<100 mm	26	32,673	3,333	6.87% (5.08–9.30)	860.02	<0.01	96.4%		
	100–400 mm	7	2,329	129	6.10% (3.92–9.51)	53.05	<0.01	84.9%		
	>400 mm	2	2083	54	2.64% (2–3.49)	0.18	−0.98	0.0%	<0.05	−0.96 (−1.80 to −0.11)
Humidity										
	20–35%	12	6,004	587	6.53% (3.64–11.69)	311.83	<0.01	96.5%		
	35–50%	12	6,468	480	6.75% (5.20–8.77)	93.65	<0.01	88.3%	>0.05	0.05 (−0.51–0.61)
	50–65%	11	24,613	2,449	5.21% (3.32–8.16)	293.07	<0.01	96.6%		
Temperature										
	<25°C	6	3,540	198	7.60% (5.53–10.43)	192.08	<0.01	95.3%		
	25–30°C	20	11,079	943	4.65% (2.84–7.62)	153.41	<0.01	92.8%		
	>30°C	9	22,466	2,375	6.55% (4.46–9.62)	681.84	<0.01	96.8%	>0.05	0.14 (−0.35–0.63)
Climate										
	Sub-tropical	28	16,559	1,309	5.87% (4.36–7.89)	847.92	<0.01	96.1%		
	Humid-sub tropical	5	19,463	2,138	6.16% (3.59–10.56)	185.89	<0.01	96.2%		
	Hot desert	2	1,063	69	10.48% (5.29–20.77)	24.68	<0.01	91.9%	>0.05	0.57 (−0.38–1.53)
Total		35	37,085	3,516	6.06% (4.67–7.87)	770.91	<0.01	96%		

CI*, confidence interval.

## 3 Results

### 3.1 Literature search results and quality assessment of the included studies

**Figure 1:**
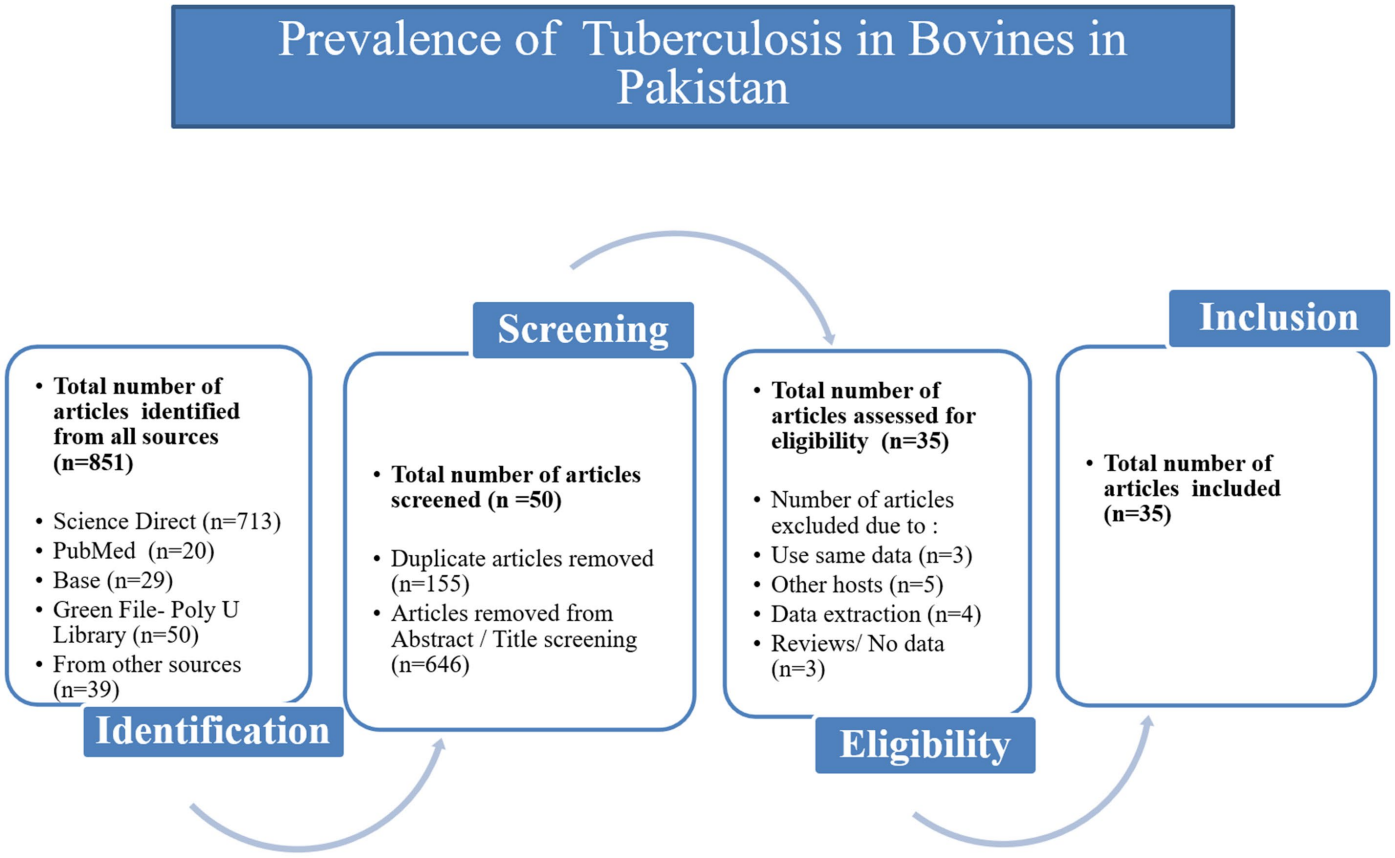
Flow diagram of the selection of eligible studies, based on the inclusion and exclusion criteria specific to this study.

From 2000 to 2024, 851 articles were screened from the four databases, Google Scholar and publications cited in the published research (Figure 1). According to inclusion and exclusion criteria, 35 articles were included in this meta-analysis (Supplementary Table S5). The score of each article was shown separately to represent its evaluation in the analysis (Table 1). The meta-analysis of the 35 studies showed an overall prevalence of bTB (6.06% [95% CI: 4.67–7.87]), with 3,516 positive cases from a total of 37,085 samples and high heterogeneity between studies.

### 3.2 Publication bias

High heterogeneity was found in the included studies (*I^2^* = 96%, *p* ≤ 0.01) (Figure 2), and PLN was used on the positive rate to make sure the total effect size data was closer to a normal distribution (Table 2). A funnel plot was used to identify publication bias. The asymmetrical scatter distribution suggests the possibility of publication bias or small sample size bias in the study (Figure 3). Publication bias was further confirmed using the Egger linear regression approach. Egger’s test (*p = 0.001*) also showed that there exists publication bias in our studies (Figure 4; Supplementary Table S3). The results of meta-analysis and publication bias of each subgroup are shown in Supplementary Figures S1–S34.

**Figure 2:**
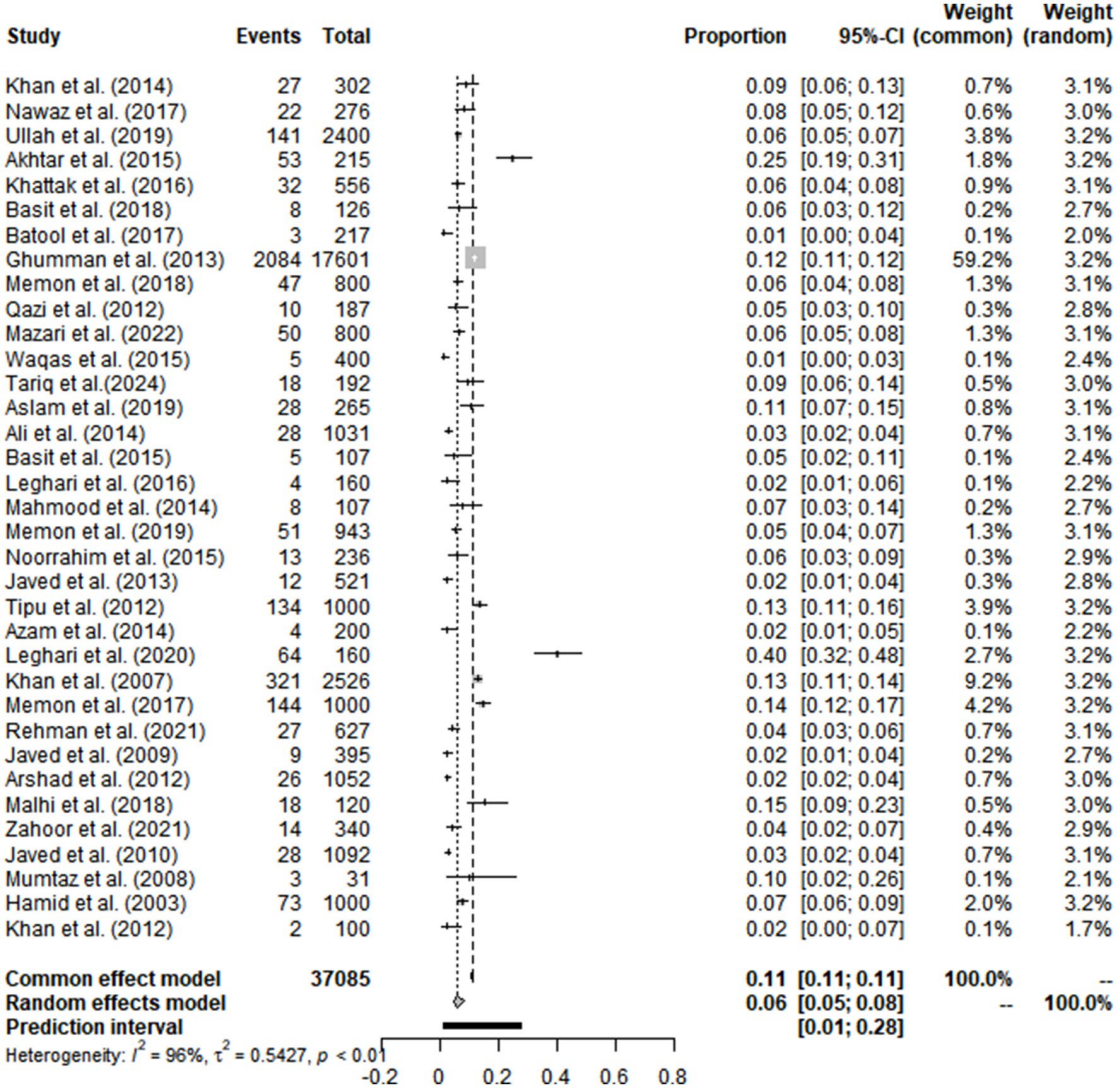
Forest plot of random effects models for bovine tuberculosis prevalence in Pakistan.

**Figure 3:**
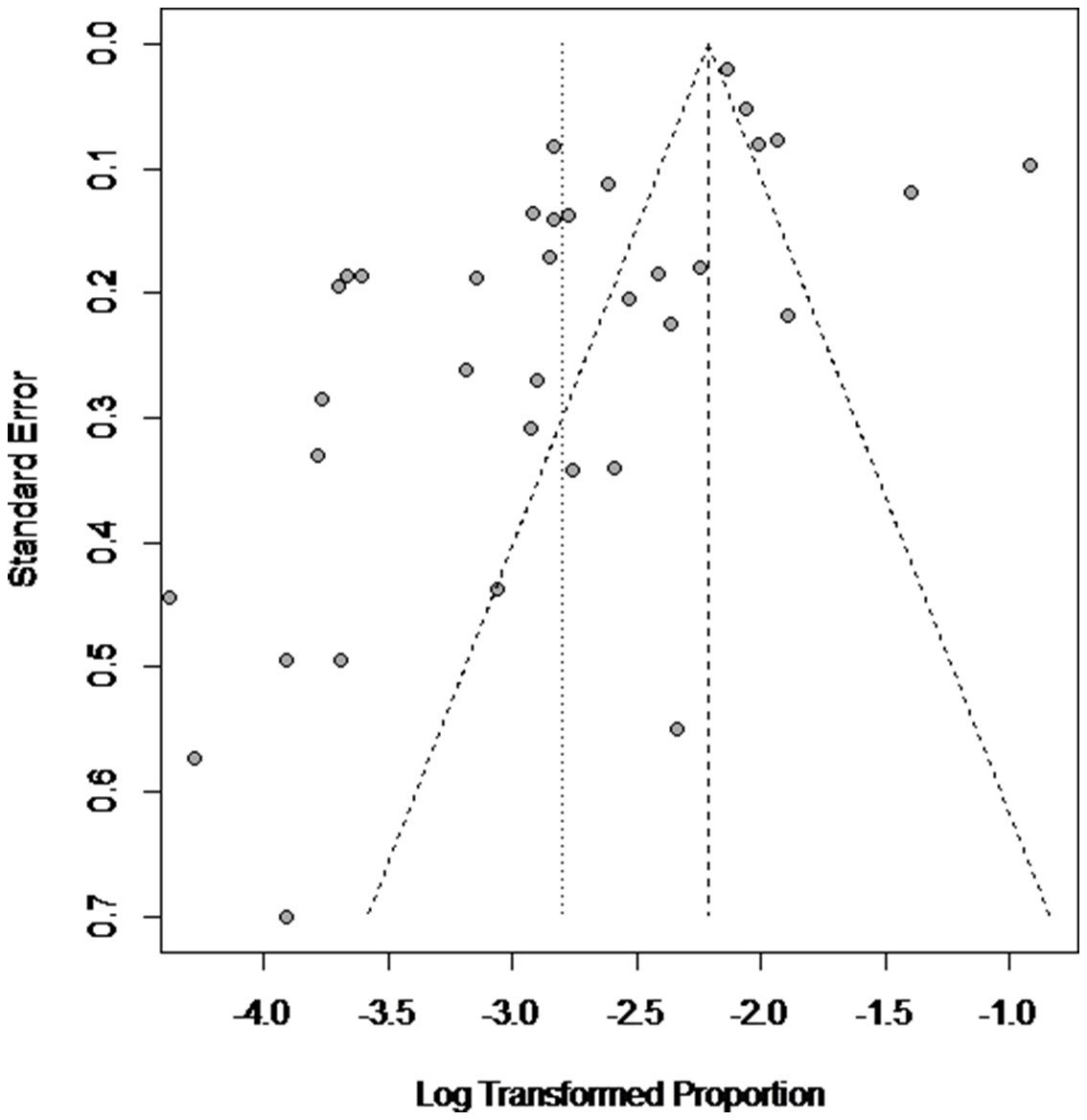
Funnel plot with pseudo 95% confidence interval limits for the examination of publication bias.

**Figure 4:**
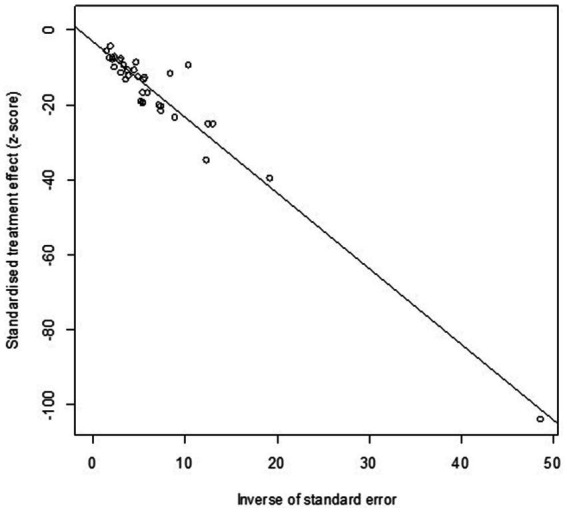
Egger’s test for publication bias.

### 3.3 Sensitivity analysis

**Figure 5:**
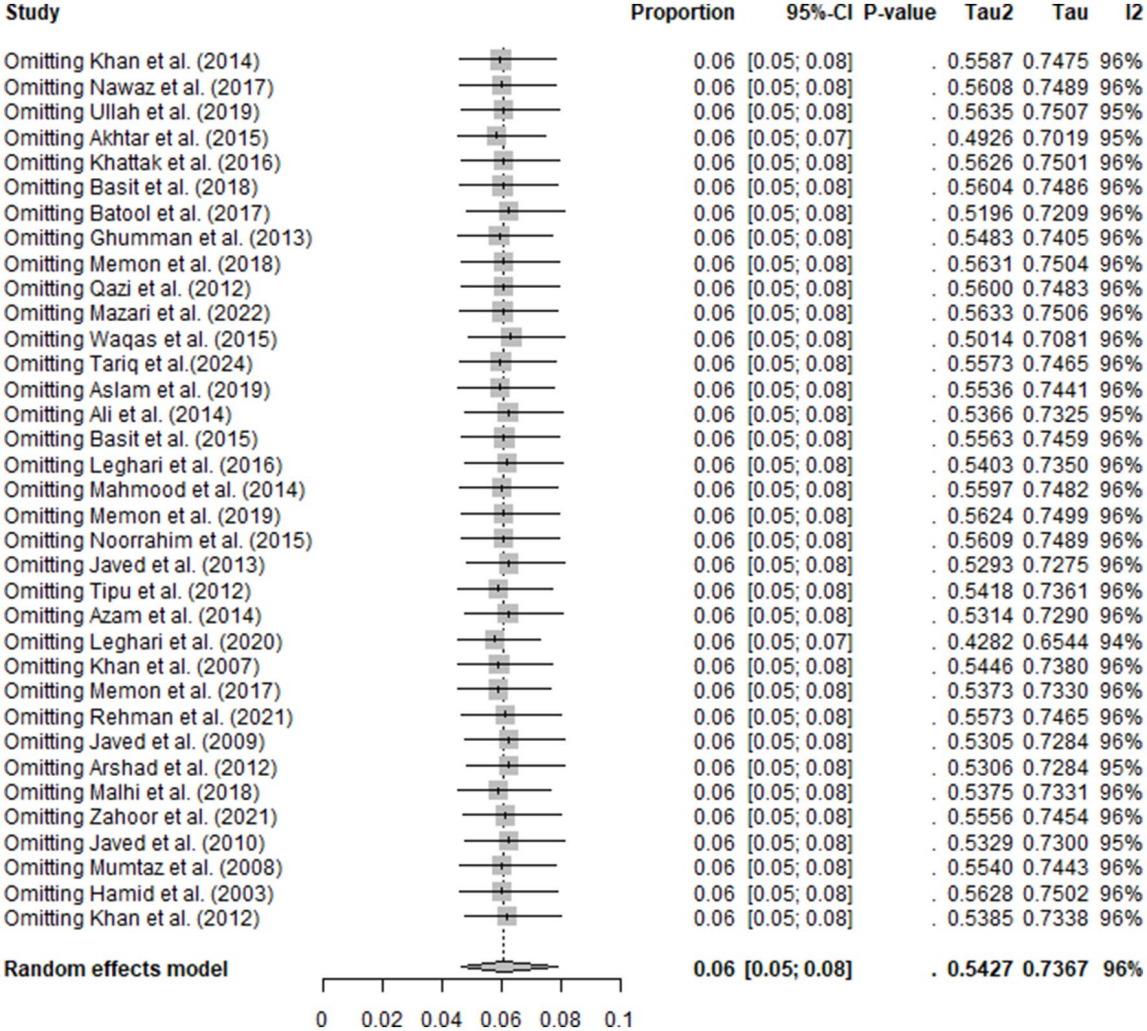
Sensitivity analysis.

Sensitivity analysis confirmed the robustness of the overall study’s findings. The omission of any single study did not affect the results, and the remaining studies gave the same results. This finding confirmed stability and reliability of the meta-analysis (Figure 5).

### 3.4 Meta-analysis of bovine tuberculosis in Pakistan

**Figure 6:**
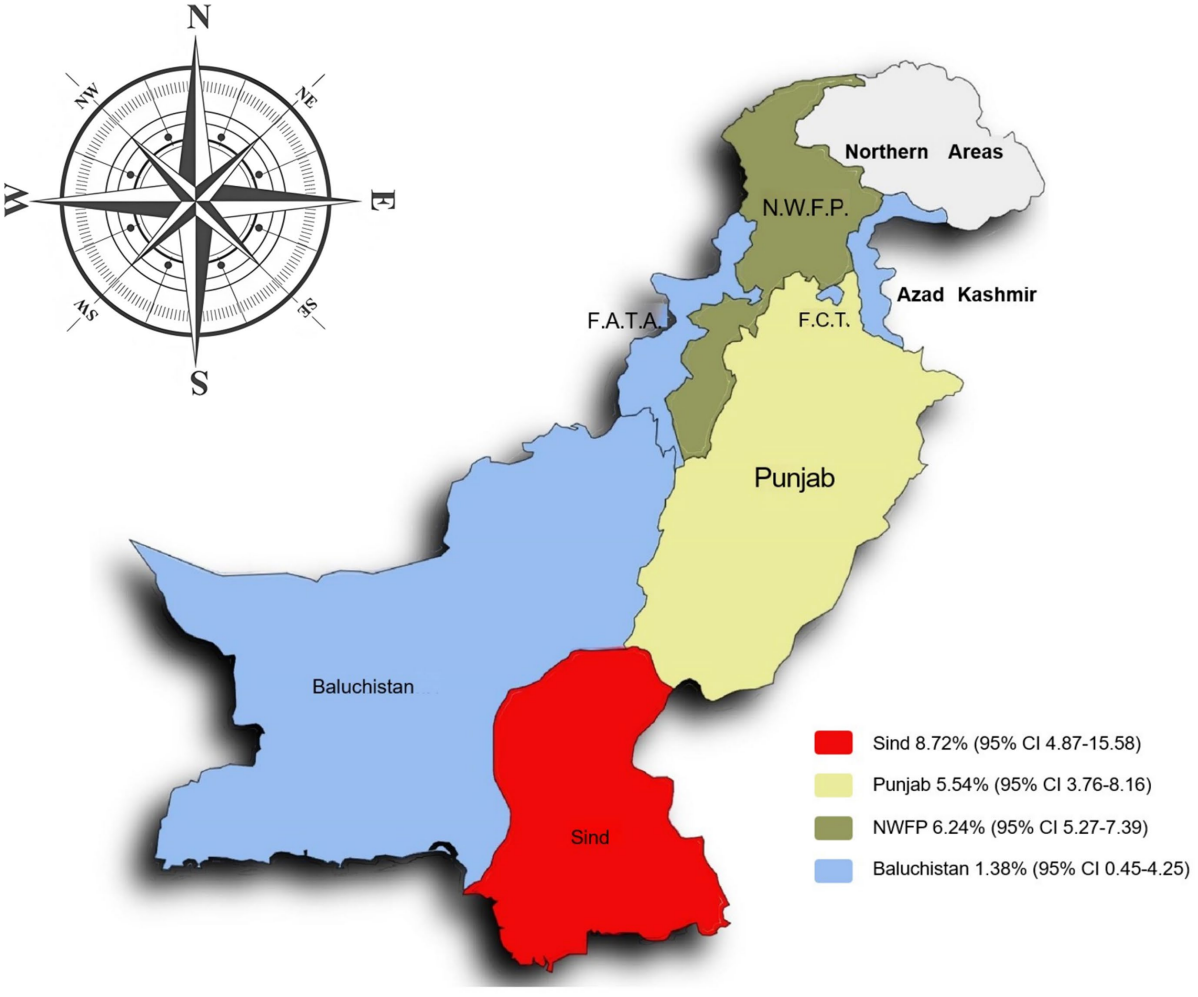
Map of bovine tuberculosis prevalence in Pakistan.

The prevalence of bovine tuberculosis (bTB) in different regions of Pakistan between 2000 and 2024 showed a significant variation. The highest prevalence was recorded in Southeast Pakistan (Sindh) at 8.72% [95% CI: 4.87–15.58]. In contrast the lowest prevalence was observed in Southwest Pakistan (Balochistan) at 1.38% [95% CI: 0.45–4.25]. However, as there is only one study available from Southwest Pakistan, the result should be interpreted with caution (Table 3; Figure 6) (52).

### 3.5 Factors associated with bovine tuberculosis

Based on subgroup analysis, the contribution of multiple parameters were assessed as risk factors for prevalence of bTB.

#### 3.5.1 Factors related to the animal

Regarding animal species, cattle were the most susceptible one, with a highest prevalence of 6.44% [95% CI: 4.04–10.26], compared to bovine 6.07% [95% CI: 4.11–8.97] and buffaloes 5.54% [95% CI: 3.13–9.81] with no significant difference. Interestingly, the animal breed as found to be a significant risk factor as the exotic breeds showed a higher prevalence (13.65% [95% CI: 5.44–34.24]) compared to local breeds (5.33% [95% CI: 3.32–8.56]) and cross-bred (9.72% [95% CI: 3.85–24.56]) (Table 3).

Concerning the gender as a risk factor, females were found to have high positive rate (6.72% [95% CI: 4.47–10.08]) comparing to males (4.41% [95% CI: 2.63–7.39]) with no significant difference.

Regarding the pregnancy as a risk factor, prevalence was found to be high in the pregnant females (10.75% [95% CI: 2.93–39.50]) compared to the non-pregnant females (8.84% [95% CI: 4.00–19.56]) with no significant difference.

Lactating animals exhibited a higher prevalence rate (9.93% [95% CI: 5.80–17.02]) compared to non-lactating animals (6.75% [95% CI: 3.90–11.67]) with no significant difference.

Concerning the body condition score (BCS) (Supplementary Table S4), the animals with fair BCS showed the highest prevalence (17.66% [95% CI: 3.63–85.88]) compared to poor BCS (13.51% [95% CI: 6.39–28.58]) and good BCS (2.43% [95% CI: 1.29–4.60]) with significant difference.

The disease prevalence was differed according to animal age. It could be cleared that, animals of age (4–6 years) showed higher prevalence (10.26% [95% CI: 6.31–16.69]) compared to animals of age (7–10 years) (8.02% [95% CI; 5.09–12.64]) and animals of age (1–3 years) (3.50% [95% CI: 2.75–4.46]) with significant difference.

#### 3.5.2 Factors related to the environment and climatic conditions

Geographical factors were found to have influence on bTB prevalence. Regarding latitude as a risk factor, regions with a low altitude (24–28°) showed higher prevalence (9.29% [95% CI: 5.56–15.53]) compared to regions of altitude 31–35° (6.24% [95% CI: 5.26–7.39]) and altitude 28–31° (5.04% [95% CI: 3.43–7.40]) with significant difference. Regarding longitude as risk factor, regions with a low longitude 66–69° showed the highest prevalence (8.21% [95% CI: 4.80–14.06]) compared to regions of longitude 69–72° (6.43% [95% CI: 5.48–7.53]) and > 72° (5.11% [95% CI: 3.50–7.44]) with no significant difference.

Studying of altitude as a risk factor revealed that, regions with a low altitude <100 m showed the highest prevalence (8.66% [95% CI: 4.05–18.52]) compared to regions with altitude >1,500 m (6.56% [95% CI: 2.79–15.40]) and altitude 100–1,500 m (5.71% [95% CI: 4.28–7.62]) with no significant difference.

The rainfall was evaluated as a risk factor, regions with low rainfall <100 mm showed highest prevalence (6.87% [95% CI; 5.08–9.30]) compared to regions with 100–400 mm rainfall (6.10% [95% CI: 3.92–9.51]) and with >400 mm rainfall (2.64% [95% CI: 2–3.49]) with significant difference.

Temperature as a risk factor was found to be a significant one, regions with an average annual temperature < 25°C had the highest prevalence (7.60% [95% CI: 5.53–10.43]) compared to regions with temperature > 30°C (6.55% [95% CI: 4.46–9.62]) and with temperature 25–30°C (4.65% [95% CI: 2.84–7.62]) with no significant difference.

Parallelly the regions of hot desert climate showed the highest prevalence (10.48% [95% CI: 5.29–20.77]) compared to regions of humid-sub tropical climate (6.16% [95% CI: 3.59–10.56]) and sub-tropical climate regions (5.87% [95% CI: 4.36–7.89]) with no significant difference (Table 4).

The regions with 35–50% humidity showed highest prevalence (6.75% [95% CI: 5.20–8.77]) compared to regions having 20–35% humidity (6.53% [95% CI: 3.64–11.69]) and regions with humidity 50–65% (5.21% [95% CI: 3.32–8.16]) with no significant difference.

#### 3.5.3 Factors related to management

The significance of herd size as a risk factor was determined, highest prevalence rate was found in larger herds having (101–500 animals) (10.59% [95% CI: 3.90–28.75]) compared to small herds having (1–50 animals) (7.09% [95% CI:4.81–10.44]) and moderate herds (51–100 animals) (2.50% [95% CI: 1.88–3.33]) with significant difference.

Long lactation period (6–10 M) was found to be significantly (6.32% [95% CI: 2.57–15.54]) high compared to lactation length (3–6 M) (7.54% [95% CI: 2.80–20.30]) and lactation length (0–3 M) (1.97% [95% CI: 1.20–3.25]).

The heavy weighted animals (400–500 kg) showed higher prevalence (7.75% [95% CI: 4.20–14.28]) compared to animals having weight (300–400 kg) (3.56% [95% CI: 1.12–11.33]) and animals with weight (200–300 kg) (2.26% [95% CI: 0.82–6.23]) with significant difference.

#### 3.5.4 Other risk factors

Regarding the study’s duration as risk factor, the time period from 2007 to 2011 showed highest prevalence (9.24% [95% CI: 6.49–13.16]) compared to 2011–2017 (5.90% [95% CI: 3.54–9.84]), and 2005–2007 (3.32% [95% CI: 1.87–5.89]) with no significant difference.

Although different methods were used for bTB, polymerase chain reaction (PCR) showed the highest prevalence (5.65% [95% CI: 3.33–9.58]), compared to the tuberculin skin test (TST) (5.61% [95% CI: 4.20–7.50]) and culture test (4.67% [95% CI: 2.50–8.72]) with no significant difference.

The sample type was an important factor to be evaluated. The highest prevalence was determined in milk samples (14.66% [95% CI: 7.38–29.11]) compared to nasal swabs (11.62% [95% CI: 5.19–26.02]) with no significant difference.

Concerning parity as a risk factor, the parity number 3–6 showed highest prevalence (14.10% [95% CI: 4.31–46.12]) compared to parity number 1–3 (7.65% [95% CI: 2.31–25.40]) with no significant difference.

Regarding quality level of studies as risk factor, it was found that, studies with higher quality scores (3–4) showed a higher prevalence (6.24% [95% CI: 4.46–8.73]) compared to quality scores (1–2) (5.74% [95% CI: 3.89–8.45]) (Table 3).

## 4 Discussion

Pakistan located in subtropical South Asia, has reported multiple cases of bTB across the country, primarily caused by *M. bovis* (53, 54). Although the majority of developed countries have successfully eliminated bTB through extensive application of test-and-slaughter programs, but the disease remains endemic in multiple areas of Africa, Asia, Latin America, and a large portion of the Middle East, posing serious public health and economic risks (40, 54). Low-and middle-income countries like Pakistan face additional challenges due to inadequate resources and incomplete data (53, 55, 56). On the human side, *M. bovis* is a neglected pathogen, excluded from the “National Guidelines for the Control of Tuberculosis in Pakistan-2019.” Despite efforts by the WHO (Supplementary Table S4) and other international organizations to tackle zoonotic TB, the country lacks an effective bTB surveillance program (57). Although small-scale studies have been conducted, national initiatives remain absent despite the availability of infrastructure from previous disease control efforts like Rinderpest eradication (56, 57).

The current meta-analysis showed an overall bTB prevalence of (6.06%) in Pakistan with highest prevalence in cattle (6.44%), as compared to buffalo (5.54%) and bovines (6.07%). These findings align with regional variations observed globally, including India (2018) (7.3%) (58), and much lower than those recorded in Ghana (19%) during (2011–2012) (59), (28%) in South Africa (2016–2017) (60), (22%) in Ethiopia (2016–2017) (61). The highest prevalence rate in Pakistan (9.24%) occurred between 2007 and 2011, significantly influenced by the devastating 2010 floods which caused severe losses to the agriculture and livestock sectors (62). Of the provinces affected by this damage, Sindh incurred (46%) of the total losses, followed by Punjab (36%), Khyber Pakhtunkhwa (8%), and Balochistan (8%). The overall estimated loss to this industry was approximately $5 billion (63). The 2010 summer floods resulted in the deaths of 274,334 domesticated animals due to lack of food and fodder, nutritional deficiencies, weakened immune systems and an increased susceptibility to diseases including bovine tuberculosis (64, 65). In Africa, increased TB prevalence is linked to flooding due to enforced contact between herds (66), and drought, which forces cattle to use communal water sources (67), and encourages large-scale movements (68). Ranking fifth in the 2019 Global Climate Risk Index (CRI), Pakistan has a high susceptibility to the impacts of climate change (69).

Detection methods in the meta-analysis primarily included the (TST, PCR and bacterial culture). PCR showed higher detection rate (5.65%) compared to the Tuberculin Skin Test (TST) (5.61%), the most preferred, and widely used diagnostic method (70) due to its cost-effectiveness and widespread availability. However, its sensitivity varies across regions and management conditions (71) with concerns about under-diagnosis and false negatives that could lead to disease re-emergence in cattle herds (16, 72). Although PCR has a comparable positive rate (5.65%), its application is limited in Pakistan due to the lack of advanced diagnostic facilities and small study sample sizes. On the other hand, the Culture test method, which is considered the gold standard test for bTB determination, requires BSL-3 laboratory-facilities that are unavailable in many developing countries. It is worth noting that molecular techniques were widely used for pathogen detection because of its high sensitivity, and less time consuming (22, 73, 74). Therefore, future researches in Pakistan should assess the effectiveness of TST in addition to PCR and culture, along with these, taking into account the need for more effective and accessible testing techniques, to increase the diagnostic accuracy and decrease false negatives (16).

In Pakistan, milk is mostly produced from bovines. So, it is important to determine the *M. bovis* status in both cattle and buffaloes to determine the bovine tuberculosis risk. Remarkably, the majority of previous studies conducted in Pakistan have focused on cattle. In contrast, the buffalo population, which constitutes a significant portion of country’s livestock and appears more susceptible to bovine tuberculosis (75), has been the subject of limited research, primarily restricted to regions such as districts of Okara and Faisalabad (76). Regarding animal species (cows vs. buffalo), the meta-analysis showed a higher prevalence in cows (6.44%) compared to buffalo and bovines. Similar results have been reported using RE model that might be due to management and biological factors (58). Higher prevalence in cows might be due to the high- density breeding environments in commercial dairy farms, where close contact between animals favors the spread of pathogens including *M. bovis*, in contrast buffaloes were raised in extensive systems with lower breeding densities, thereby reducing risk transmission (66, 77).

A high prevalence of bTB (11.7%) has been shown in several dairy cattle farms of Punjab, Pakistan (78). Cattle are biologically susceptible to *M. bovis,* frequently resulting in subclinical infections and transmission occurs either through inhalation or oral route (79). Milk samples showed the highest prevalence (14.66%) in our study indicating probability of zoonotic risk of transmission, especially in areas where dairy products are improperly handled and not pasteurized (80). Key factors contributed to the spread of bovine tuberculosis included consuming raw milk, close contact with animals, and poor hygiene standards on animal farms (81).

The primary way that bovine tuberculosis (bTB) is spread from cow to calf is through the consumption of contaminated milk or colostrum (43). *M. bovis* primarily affects the mammary glands of cattle and buffaloes, sub-clinically infected cows usually shed *M. bovis* at concentrations of about 10^3^ colony forming unit (cfu/ml) (82). So, the bacteria are more likely to be excreted in the milk of the infected animals frequently without apparent clinical symptoms (82, 83). Depending on the immunological response, *M. bovis* can potentially spread by aerosol inhalation, which allows it to enter the lungs and be absorbed by alveolar macrophages, resulting in localized or systemic infection. Effective immunity blocks the infection and stops active shedding, but impaired immunity causes persistent lesions and active pulmonary tuberculosis, which spreads *M. bovis* through aerosol, feces, mucus, urine and milk (84). To determine the prevalence of *M. bovis* in milk, various diagnostic methods were employed in previous studies. The overall prevalence of *M. bovis* in milk samples was (5% [95% CI; 3–7%]). Among cows that tested positive for the tuberculin skin test (TST), the prevalence increased to (8% [95% CI; 4–13%]) likely due to the association between positive TST results and active or latent infections. Regardless of the herd TST infection status, the prevalence of *M. bovis* in bulk tank milk (BTM) was estimated at (5% [95% CI; 0–21%]) (85). These findings highlight milk transmission as an important risk factor. However, a separate meta-analysis of milk as a transmission factor was not conducted in this study due to the limited number of studies with sufficient data.

In the current study, prevalence rate was higher in females (6.72%) than in males (4.4.1%), which might be attributed to the differences in production systems and stress factors associated with females as pregnancy, parturition and lactation (86, 87). Males are closely related to beef farms and females are found on dairy farms (12). Therefore, prevalence might be higher in females due to the large number of samples collected particularly from dairy herds (60). However, some studies reported high prevalence in males as they were mainly used for oxen and kept in herds for longer time thus increasing their contact with infected herds (88). Management practices also differ between genders in both developing and developed countries. Due to close contact during milking and calving, dairy cows were more susceptible to bovine tuberculosis than males and they also achieve maturity earlier (89). This emphasizes the significance of routine bTB testing and efficient herd management to prevent the spread of disease within dairy herds.

Exotic breeds such as Holstein-Friesians were more prone to the disease and showed higher prevalence (13.65%) as compared to local and cross-bred due to difference in genetic resistance and adaptation to local environmental conditions (22). Moreover, research suggested association between the *TauT* gene (Taurine Transporter) in Holstein-Friesian cattle and bTB susceptibility (90), its presence leads to taurine deficiency and ultimately affecting immune system (91). Exotic breeds have been bred for high milk production in temperate climates; these breeds may lack the genetic adaptations for disease resistance prevalent in sub-tropical and tropical regions like Pakistan. This stress resulted in impairment of immunity, making them more vulnerable to bTB (92). The obtained results were aligned with previous studies showed a higher prevalence of bTB in exotic cattle (93, 94).

Age was the main individual risk factor in various studies in both developed and developing countries. Age and weight were interrelated factors contributed to higher prevalence of bovine tuberculosis in livestock (95). The prevalence of *M. bovis* was higher in animals aged 4–6 years (10.26%), followed by in animals aged 7–10 years (8.60%). Similarly, animals with an increased weight showed a prevalence of (7.75%). These patterns may result from the long incubation period and slow progression of the disease to detectable levels (96). The later decline in prevalence could be due to the development of an allergic state or a higher mortality rate among infected animals in the advanced stage of disease (97). Older animals often gain more weight, particularly due to increased fat deposits, which can lead to chronic-low grade inflammation and a weakened immune response. This impairment results in significantly higher rates of seropositivity. Animals may get infected at a young age but at adult stage they express disease clinically (98). Mycobacteria have the ability to remain in latent state for longer periods before being reactivated at old age (99). Scientists have not yet confirmed whether cattle can harbor a true dormant state of infection (100). Developing of experimental latency models in cattle is necessary to evaluate their implications, including the underdiagnosis of the disease, especially in the developed countries (89). Similar results have been found in studies that bTB infection increases with age, with older heavier animals more susceptible to disease (95).

In the current analysis, larger herds (101–500 animals) had more prevalence (10.59%) compared to the small ones. The same observation was previously recorded (101), especially with poor management practices (102). Factors such as defective ventilation (94%), improper waste disposal system (94.18%), cattle in same premises (92.59%) and poor floor sanitation (77.77%) were directly or indirectly related to the spread of bTB through coughing (103). As the dairy farmers continuously expand their herd size to increase farm yield, leading to overcrowding thus causing increased risk of animal-to-animal transmission (104).

In the present study, pregnant animals showed the highest prevalence (10.75%) compared to the non-pregnant ones, which correlate with other studies suggesting that pregnant cattle are more susceptible to infection (70). This could be due to many factors including the physiological, and hormonal changes during pregnancy which might suppress the immune system making them more susceptible at that time (105).

The study identified an increased prevalence of bTB (9.93%) in lactating animals, especially those with higher parity and longer lactation periods. The increased production stress in dairy cows endure and the gathering of cows during milking might increase the risk of disease transmission (106). Similar findings have been reported in previous studies that higher prevalence was observed in lactating as compared to non-lactating ones, mostly due to increased stress of high milk production (70, 107, 108). Furthermore, during lactation adult females may suffer from nutritional deficiency that can be a predisposing factor for bovine tuberculosis and might reduce the immunity against diseases (109, 110). Due to weakened immunity, animals become more vulnerable to infections especially when producing large quantities of milk over extended periods and through multiple pregnancies (111). Hence, the disease prevalence rate is directly proportional to the increase in milk production (112).

Our findings align with studies showing that animals with good BCS were less susceptible to bTB infection compared to those with fair (36, 61) or poor scores (113, 114). In contrast, some studies suggested a link between good BCS and high prevalence. This might be due to factors as increased milk production in well-nourished animals leading to weakened immune system thereby increasing infections as bTB (115).

In terms of regional and provinces sub-groups, Sindh group in southeastern of Pakistan, showed the highest prevalence (8.72%) of bTB infection. Flood-prone areas have been shown to favor the environmental persistence of other *Mycobacteria* species (116), because of the high moisture content that helps *M. bovis* to survive longer (117). The geographical regions with varied climates directly impact the environmental persistence and transmission dynamics of *M. bovis* (82, 118). Low altitude and decreased rainfall showed significant differences and highest prevalence in the meta-analysis. Sindh is also located at low altitude (<100 m) and latitude (24–28^o^), consisting of plain and flat areas with low rainfall (<100 mm) and hot climate. These conditions may facilitate bacterial survival and transmission, along with congregation of animals due to fewer water sources. Additionally, harsh environmental conditions, poor nutrition, and stress can weaken the animals’ immune systems, which increases their vulnerability to disease (119). All of these factors may contribute to the higher prevalence of *M. bovis* in this region.

The peri-urban areas, transitional zones between rural and urban settings, were susceptible to bTB due to presence of both extensive and intensive farming systems. Intensive livestock farming, poor sanitary conditions in rural farms and close contact at water farms favor the ideal environment for *M. bovis* infection (120). An increased incidence of bTB occurrences was related to intensive management approaches that enhance cattle-to-cattle contact within a herd (121). Farms and abattoirs having regulated environments where animals are rigorously screened for diseases, mostly implement strict biosecurity measures and more disease control programs including controlled breeding ones and vaccination schedules, which help in preventing the spread of disease as compared to less regulated environments in rural, urban and town settings. These findings were consistent with previous studies reporting higher prevalence in peri-urban, rural and urban settings (10, 122).

While our study included 24 high-quality and 11 average-quality articles. In most of the average articles, prevalence was low due to lack of clear sampling time and random sampling descriptions. The researchers must provide detailed relevant information about risk factors during epidemiological investigations thus providing reliable scientific data and effective follow-up research on bovine tuberculosis.

Our meta-analysis has some limitations including mainly the limited used databases. Accordingly, some eligible studies might be excluded. Also, some risk factors were examined in small number of studies, which could result in potentially unstable results due to small study effects. Most of included studies were from Northeast Pakistan, with only one study from Southwest Pakistan. Lastly, in interpreting the results of this meta-analysis, it is important to acknowledge the limitations associated with the use of univariate meta-regression.

We acknowledge the limitations of performing only univariable meta-regression in this study. While this method provides information on the independent association of each variable with tuberculosis prevalence, it does not account for confounding effects or correlations between variables. As such, results should be interpreted with caution, since associations found in isolation may become non-significant when adjusted for other variables in a multivariable model. A key limitation is the risk of multicollinearity, some study variables may be correlated, potentially leading to an over or underestimation of their true effects. Without a multivariable approach, it is difficult to distinguish direct effects from those influenced by confounding. Moreover, univariable meta-regression does not allow adjustment for multiple factors simultaneously, which can result in biased estimates, especially when variables interact or jointly affect tuberculosis prevalence.

These issues could be mitigated using a multivariable meta-regression model, which would control for key variables and reduce the impact of multicollinearity. However, due to limitations such as missing data and the small number of included studies, applying such a model risked overfitting and unreliable estimates. Given these constraints, we used univariable analyses as an initial step, with the understanding that future studies with larger datasets should employ multivariable meta-regression for more robust analysis. Despite these limitations, we interpreted our findings cautiously to minimize bias and provide meaningful preliminary insights. We emphasize that the associations reported here should be validated in future research using enhanced methodologies, including multivariable meta-regression, to improve result reliability.

## 5 Conclusion

This meta-analysis reveals the significant prevalence and risk factors associated with bTB in Pakistan, emphasizing its implications for livestock management, public health and economic sustainability. The overall prevalence of bTB (6.06%) aligns with regional trends but it is notably higher in certain areas and specific sub-groups such as dairy cows, lactating, pregnant animals, exotic breeds, and larger herds. Factors such as intensive farming, poor management practices and climate-induced stressors including flooding and drought, exacerbate the disease’s persistence and spread. The high prevalence of *M. bovis* in milk samples underscores the zoonotic risk of transmission in areas lacking pasteurization and proper hygiene standards. The study also highlights diagnostic challenges, with PCR showing comparable detection rates to TST but with limited use due to inadequate infrastructure. The gold standard culture test remains underutilized due to resource constraints, emphasizing the urgent need for accessible and accurate diagnostic tools.

Pakistan faces significant challenges in eradicating and controlling the bTB due to a lack of data, inadequate diagnostic and treatment facilities and limited awareness about the disease. Adopting a “One Health Approach” is crucial for effective control, integrating the livestock, agriculture, public health and food security sectors. Priorities include establishing comprehensive surveillance programs, improving diagnostic methods, focusing on underrepresented regions and buffalo populations, and addressing management-related issues. Strengthened biosecurity measures, effective herd management and farmer education are essential to reduce transmission. Coordinated national and regional efforts, supported by international organizations, are important for developing and implementing policies to protect public health and livestock industry.

## Data Availability

The raw data supporting the conclusions of this article will be made available by the authors, without undue reservation.
