# Supplementary material for: Prevalence of tuberculosis in bovines in Pakistan during 2000–2024: a systematic review and meta-analysis
Source: Front Vet Sci. 2025 Apr 17;12:1525399. doi: 10.3389/fvets.2025.1525399 (PMC12043705; doi:10.3389/fvets.2025.1525399)
Supplement: Supplementary file 1 [file Data_Sheet_1.docx]

**Table S1** PRISMA Checklist item.

| **Section/topic** | **#** | **Checklist item** |
| --- | --- | --- |
| **TITLE** |  |  |
| Title | 1 | Prevalence of tuberculosis in bovines in Pakistan during 2000-2024: A systemic review and meta-analysis |
| **ABSTRACT** |  |  |
| Structured summary | 2 | Provide a structured summary including, as applicable: background; objectives; data sources; study eligibility criteria, participants, and interventions; study appraisal and synthesis methods; results; limitations; conclusions and implications of key findings; systematic review registration number. |
| **INTRODUCTION** |  |  |
| Rationale | 3 | Describe the rationale for the review in the context of what is already known. |
| Objectives | 4 | Provide an explicit statement of questions being addressed with reference to participants, interventions, comparisons, outcomes, and study design (PICOS). |
| **METHODS** |  |  |
| Protocol and registration | 5 | Indicate if a review protocol exists, if and where it can be accessed (e.g., Web address), and, if available, provide registration information including registration number. |
| Eligibility criteria | 6 | Specify study characteristics (e.g., PICOS, length of follow-up) and report characteristics (e.g., years considered, language, publication status) used as criteria for eligibility, giving rationale. |
| Information sources | 7 | Describe all information sources (e.g., databases with dates of coverage, contact with study authors to identify additional studies) in the search and date last searched. |
| Search | 8 | Present full electronic search strategy for at least one database, including any limits used, such that it could be repeated. |
| Study selection | 9 | State the process for selecting studies (i.e., screening, eligibility, included in systematic review, and, if applicable, included in the meta-analysis). |
| Data collection process | 10 | Describe method of data extraction from reports (e.g., piloted forms, independently, in duplicate) and any processes for obtaining and confirming data from investigators. |
| Risk of bias in individual studies | 12 | Describe methods used for assessing risk of bias of individual studies (including specification of whether this was done at the study or outcome level), and how this information is to be used in any data synthesis. |
| Summary measures | 13 | State the principal summary measures (e.g., risk ratio, difference in means). |
| Synthesis of results | 14 | Describe the methods of handling data and combining results of studies, if done, including measures of consistency (e.g., I^2^) for each meta-analysis. |
| Risk of bias across studies | 15 | Specify any assessment of risk of bias that may affect the cumulative evidence (e.g., publication bias, selective reporting within studies). |
| Additional analyses | 16 | Describe methods of additional analyses (e.g., sensitivity or subgroup analyses, meta-regression), if done, indicating which were pre-specified. |
| **RESULTS** |  |  |
| Study selection | 17 | Give numbers of studies screened, assessed for eligibility, and included in the review, with reasons for exclusions at each stage, ideally with a flow diagram. |
| Study characteristics | 18 | For each study, present characteristics for which data were extracted (e.g., study size, PICOS, follow-up period) and provide the citations. |
| Risk of bias within studies | 19 | Present data on risk of bias of each study and, if available, any outcome level assessment (see item 12). |
| Results of individual studies | 20 | For all outcomes considered (benefits or harms), present, for each study: (a) simple summary data for each intervention group (b) effect estimates and confidence intervals, ideally with a forest plot. |
| Synthesis of results | 21 | Present results of each meta-analysis done, including confidence intervals and measures of consistency. |
| Risk of bias across studies | 22 | Present results of any assessment of risk of bias across studies (see Item 15). |
| Additional analysis | 23 | Give results of additional analyses, if done (e.g., sensitivity or subgroup analyses, meta-regression [see Item 16]). |
| **DISCUSSION** |  |  |
| Summary of evidence | 24 | Summarize the main findings including the strength of evidence for each main outcome; consider their relevance to key groups (e.g., healthcare providers, users, and policy makers). |
| Limitations | 25 | Discuss limitations at study and outcome level (e.g., risk of bias), and at review-level (e.g., incomplete retrieval of identified research, reporting bias). |
| Conclusions | 26 | Provide a general interpretation of the results in the context of other evidence, and implications for future research. |
| **FUNDING** |  |  |
| Funding | 27 | Describe sources of funding for the systematic review and other support (e.g., supply of data); role of founders for the systematic review. |

*From:* Moher D, Liberati A, Tetzlaff J, Altman DG, The PRISMA Group (2009). Preferred Reporting Items for Systematic Reviews and Meta-Analyses: The PRISMA Statement. PLoS Med 6(6): e1000097. doi:10.1371/journal.pmed1000097

For more information, visit: **www.prisma-statement.org**.

**Table S2.** The code in R for this meta-analysis.

| Logarithmic conversion (PLN) | rate<-transform [m1, log=log(event/n)];  shapiro.test(rate$log) |
| --- | --- |
| Logit transformation (PLOGIT) | rate<-transform {m1, logit=log[(event/n)/(1-event/n)]};  shapiro.test(rate$logit) |
| Arcsine transformation (PAS) | rate<-transform {m1, arcsin.size=asin[sqrt(event/(n+1))]};  shapiro.test(rate$arcsin) |
| Double-arcsine transformation (PFT) | rate<transform {m1, darcsin=0.5*[asin(sqrt(event/(n+1)))+asin((sqrt(event+1)/(n+1)))]};  shapiro.test (rate$darcsin) |
| No transformation (PRAW) | rate<-transform [m1, r= event/n];  shapiro.test(rate$r) |

| Forest plots | forest [meta1, xlim=c (-0.2, 0.8)] |
| --- | --- |
| Funnel chart | funnel (meta1) |
| Egger's test | metabias (meta1, method="linreg") |
| The sensitivity analysis | Metainf (meta1, pooled = "random") forest (metainf (meta1, pooled = "random"), xlim=c(0, 0.4)) |
| Subgroup analysis | meta1<-metaprop(event, n, study, data=rate, sm="PLN", incr=0.5, allincr=TRUE, addincr=FALSE, title="", byvar= subgroup title, print.byvar=TRUE) |
| Meta-regression analysis | metareg (meta1, ~covariate title) |

**Table S3.** Egger’s test for publication bias

| Bias | se. bias | T | df | p-value |
| --- | --- | --- | --- | --- |
| -3.30 | 0.9104 | -3.63 | 33 | 0.0010 |

**Table S4.** List of abbreviations

| Terminology | Abbreviation |
| --- | --- |
| Bovine Tuberculosis  *Mycobacterium tuberculosis* complex  *Mycobacterium bovis*  World Organization for Animal Health  European Union | bTB  MTB  *M. bovis*  WOAH  EU |
| World Health Organization | WHO |
| Preferred Reporting Items for Systematic Reviews and Meta-Analyses  Grading of Recommendations, Assessment, Development and Evaluation Method  Original rate  Logarithmic conversion  Logit transformation  Arsenic transformation  Double arsenic transformation  Climate Risk Index | PRISMA  GRADE  PRAW  PLN  PLOGIT  PAS  PFT  CRI |
| Body Condition Score  Infected Milk  Bulk Tank Milk | BCS  IM  BTM |

**Table S5.** Included studies and quality scores

|  | **Reference ID** | **No. tested** | **No. positive** | **Prevalence** | **Random sampling or not** | **Detection method clear or not** | **Detailed Sampled method or not** | **Sample time clear or not** | **Four or more risk factors or not** | **Score** | **Study Quality** |
| --- | --- | --- | --- | --- | --- | --- | --- | --- | --- | --- | --- |
| 1 | Khan et al. (2014) | 302 | 27 | 0.0894% | Y | Y | Y | Y | N | 4 | High |
| 2 | Nawaz et al. (2017) | 276 | 22 | 0.0797% | N | Y | N | N | Y | 2 | Medium |
| 3 | Ullah et al. (2019) | 2400 | 141 | 0.05870% | N | Y | N | Y | Y | 3 | High |
| 4 | Akhtar et al. (2015) | 215 | 53 | 0.246% | N | Y | Y | N | Y | 3 | High |
| 5 | Khattak et al. (2016) | 556 | 32 | 0.0575% | Y | Y | N | Y | Y | 4 | High |
| 6 | Basit et al. (2018) | 126 | 8 | 0.06340% | Y | Y | Y | N | N | 3 | High |
| 7 | Batool et al. (2017) | 217 | 3 | 0.01380% | Y | Y | Y | N | N | 3 | High |
| 8 | Ghumman et al. (2013) | 17601 | 2084 | 0.1184% | N | Y | N | Y | N | 2 | Medium |
| 9 | Memon et al. (2018) | 800 | 47 | 0.05870% | N | Y | Y | N | N | 2 | Medium |
| 10 | Qazi et al. (2012) | 187 | 10 | 0.0534% | N | Y | N | N | N | 1 | Low |
| 11 | Mazari et al. (2022) | 800 | 50 | 0.0625% | N | Y | Y | N | Y | 3 | High |
| 12 | Waqas et al. (2015) | 400 | 5 | 0.0125% | N | Y | Y | N | N | 2 | Medium |
| 13 | Tariq et al. (2024) | 192 | 18 | 0.09375% | Y | Y | N | N | Y | 3 | High |
| 14 | Aslam et al. (2019) | 265 | 28 | 0.1056% | N | Y | Y | N | Y | 3 | High |
| 15 | Ali et al. (2014) | 1031 | 28 | 0.0271% | Y | Y | N | Y | N | 3 | High |
| 16 | Basit et al. (2015) | 107 | 5 | 0.0467% | N | Y | Y | N | N | 2 | Medium |
| 17 | Leghari et al. (2016) | 160 | 4 | 0.025% | Y | N | Y | N | Y | 3 | High |
| 18 | Mehmood et al. (2014) | 107 | 8 | 0.0747% | N | Y | Y | N | Y | 3 | High |
| 19 | Memon et al. (2019) | 943 | 51 | 0.054% | N | N | Y | Y | Y | 3 | High |
| 20 | Noorrahim et al. (2015) | 236 | 13 | 0.0551% | Y | Y | Y | N | N | 3 | High |
| 21 | Javed et al. (2013) | 521 | 12 | 0.023% | N | Y | N | Y | Y | 3 | High |
| 22 | Tipu et al. (2012) | 1000 | 134 | 0.134% | Y | Y | Y | Y | N | 4 | High |
| 23 | Azam et al. (2014) | 200 | 4 | 0.02% | Y | Y | Y | N | N | 3 | High |
| 24 | Leghari et al. (2020) | 160 | 64 | 0.4% | Y | Y | Y | N | Y | 4 | High |
| 25 | Khan et al. (2007) | 2526 | 321 | 0.1272% | N | Y | N | N | Y | 2 | Medium |
| 26 | Memon et al. (2017) | 1000 | 144 | 0.144% | Y | Y | N | N | Y | 3 | High |
| 27 | Rehman et al. (2021) | 627 | 27 | 0.043% | N | Y | N | N | Y | 2 | Medium |
| 28 | Javed et al. (2009) | 395 | 9 | 0.0227% | N | Y | N | Y | Y | 3 | High |
| 29 | Arshad et al. (2012) | 1052 | 26 | 0.0247% | Y | Y | N | Y | Y | 4 | High |
| 30 | Malhi et al. (2018) | 120 | 18 | 0.15% | N | Y | N | Y | Y | 3 | High |
| 31 | Zahoor et al. (2021) | 340 | 14 | 0.041% | Y | Y | N | N | N | 2 | Medium |
| 32 | Javed et al. (2010) | 1092 | 28 | 0.0256% | N | Y | N | Y | Y | 3 | High |
| 33 | Mumtaz et al. (2008) | 31 | 3 | 0.0967% | N | Y | Y | N | N | 2 | Medium |
| 34 | Hamid et al. (2003) | 1000 | 73 | 0.073% | Y | Y | Y | N | N | 3 | High |
| 35 | Khan et al. (2012) | 100 | 2 | 0.02% | N | Y | Y | N | N | 2 | Medium |

**References**

1. Khan, J., Ayaz, S., AbdElsalam, N. M., Ullah, R. R., & Shah, T. Prevalence of tuberculosis in buffalo and cattle. J Pure Appl Microbiol. 2014;8:721-726.
2. Nawaz, S., Qureshi, M. S., Khan, F. M., & Islam, Z. Prevalence and epidemiological parameters of bovine tuberculosis in cattle and buffaloes in district Peshawar, Pakistan.
3. Ullah, A., Khattak, U. S., Ayaz, S., Qureshi, M. S., Khan, I., Jan, I. U., ... & Sohail, M. L. Bovine tuberculosis (bTB): prevalence and associated risk factors in large ruminants in the central zone of Khyber Pakhtunkhwa, Pakistan*.* Pak J Zool. 2019;51(1):127-133. [doi.org/10.17582/journal.pjz/2019.51.1.127.133](http://dx.doi.org/10.17582/journal.pjz/2019.51.1.127.133).
4. Akhtar, F., Javed, M. T., Khan, M. N., Akhtar, P., Hussain, S. M., Aslam, M. S., & Cagiola, M. The use of PCR technique in the identification of Mycobacterium species responsible for bovine tuberculosis in cattle and buffaloes in Pakistan. Trop Anim Health Prod. 2015;47:1169-1175. doi.org/10.1007/s11250-015-0844-1
5. Khattak, I., Mushtaq, M. H., Ahmad, M. U. D., Khan, M. S., Chaudhry, M., & Sadique, U. Risk factors associated with Mycobacterium bovis skin positivity in cattle and buffalo in Peshawar, Pakistan.  Trop Anim Health Prod. 2016; 48:479-485. doi.org/10.1007/s11250-015-0976-3
6. Basit, A., Hussain, M., Shahid, M., Ayaz, S., Rahim, K., Ahmad, I., ... & Ali, T. Occurrence and Risk Factors Associated with Mycobacterium tuberculosis and Mycobacterium bovis in Milk Samples from North East of Pakistan. Pak Vet J. 2018;38(2):199-203. doi.org/10.29261/pakvetj/2018.038.
7. Batool, B. T., Tareen, A., Ahmed, S. S., Ejaz, H., Kakar, M. A., Rehman, S. A., ... & Shahwani, M. N. Prevalence of zoonotic tuberculosis and brucellosis in animals of Quetta and Pishin Districts, Balochistan. Pak J Zool. 2017;49(1):387-389. [doi.org/10.17582/journal.pjz/2017.49.1.sc4](http://dx.doi.org/10.17582/journal.pjz/2017.49.1.sc4).
8. Ghumman, M. A., Manzoor, A. W., Naz, S., Ahmad, R., & Ahmad, R. Prevalence of tuberculosis in cattle and buffalo at various livestock farms in Punjab. IJVMR: Research and Reports. 2013:1-4.
9. Memon, M. R., Bhutto, A. L., Memon, M. I., Khatri, P., & Baloch, J. A. Prevalence of Bovine tuberculosis in slaughtering animals at selected municipal slaughter houses: its impact on public health: Department of Veterinary Medicine, Sindh Agriculture University, Tandojam, Pakistan. PJAAEVS. 2018;34(2):168-175.
10. Qazi, I. H., Lochi, G. M., Mandan, A. H., Shah, I. A., Korejo, R. A., Kalhoro, A., ... & Kachiwal, A. B. Prevalence of Bovine Tuberculosis in Rural Areas of District Tando Allahyar. IJAVMS. 2012;6(5):345-348.
11. Mazari, M. Q., Kalhoro, D. H., Baloch, H., Parveen, F., Abro, S. H., Buriro, R., ... & Soomro, A. A. Prevalence and risk factors of bovine tuberculosis in cattle and dairy farm workers in Mirpurkhas and Badin districts of Sindh, Pakistan. Pak J Zool. 2022;1-8. [doi.org/10.17582/journal.pjz/20190915060930](https://dx.doi.org/10.17582/journal.pjz/20190915060930)
12. Waqas, A., Javed MT., K. Ashfaque, Mehwish Q. An Abattoir Based Study on Brucellosis, Bovine Tuberculosis and Paratuberculosis in Buffaloes and Cattle at Faisalabad, Pakistan. Int J Vet Health Sci Res. 2015;3(1):34-38.
13. Tariq, A., Aslam, A., Tipu, Y., Ahmad, M., Sultan, R., & Anjum, A. (2024). A preliminary study on prevalence of bovine tuberculosis in cattle and buffalo in outskirts of Lahore, Pakistan. Wayamba J Anim Sci. 2024:1518-1526.
14. Aslam, M. S., Javed, M. T., Khan, A., & Iqbal, Z. Bacterial and PCR based diagnosis of naturally occurring bovine tuberculosis in cattle and buffaloes. Pak J Agric Sci. 2019;56(2):481-487.
15. Ali, S., Akhtar, R., Younus, M., Saleem, G., Nisa, Q. U., & Zahid, B. Comparative trends of bovine tuberculosis in cattle and buffalo population around Lahore, Pakistan. Eur J Environ Ecol. 2014;1(1):7-11.
16. Basit, A., Hussain, M., Ayaz, S., Shahid, M., Rahim, K., Ahmad, I., ... & Gul, N. Isolation and identification of Mycobacterium bovis and Mycobacterium tuberculosis from animal tissues by conventional and molecular method. Indian J Anim Res. 2015;49(5):687-693.
17. Leghari, A., Kamboh, A. A., Dewani, P., Abro, S. H., Umrani, A. P., Malhi, K. K., ... & Shah, J. M. Isolation of Mycobacterium bovis from milk and nasal discharge samples of cattle from Hyderabad and Tando Allahyar districts. J Anim Health Prod. 2016;4(4):105-110. doi.org/10.14737/journal.jahp/2016/4.4.105.110
18. Mahmood, F., Khan, A., Hussain, R., & Khan, I. A. Molecular based epidemiology of bovine pulmonary tuberculosis–a mortal foe. Pak Vet J. 2014;34(2):185-188.
19. Mujeeb-ur-Rahman Memon, A. L., Bhutto, M. G. S., Baloch, J., Leghari, R. A., & Soomro, S. A. 4. Prevalence and pathological lesions of bovine tuberculosis assessment through routine procedures of meat inspection in infected cattle in Karachi metropolitan corporation abattoirs.  Pure Appl Biol. 2019;8(3):1909-1918. [doi.org/10.19045/bspab.2019.80134](https://dx.doi.org/10.19045/bspab.2019.80134).
20. Noorrahim, M. S. K., Shahid, M., Shah, A., Shah, M., & Rafiullah, H. A. Prevalence of tuberculosis in livestock population of district Charsadda by Tuberculin Skin Test (TST). J Entomol Zool Stud. 2015;2:15-19.
21. Javed, M. T., Wasiq, M., Farooqi, F. A., Shahid, A. L., Kausar, R., & Cagiola, M. Brief communication (Original). Certain risk factors associated with positive SCCIT test for tuberculosis in cattle at two cities in Pakistan. Asian Biomed. 2013;7(2):267-274.

[doi.org/10.5372/1905-7415.0702.175](https://doi.org/10.5372/1905-7415.0702.175)

1. Tipu, M. Y., Chaudhary, Z. I., Younus, M., & Rabbani, M. A cross sectional study of Mycobacterium bovis in dairy cattle in and around Lahore city, Pakistan. Pak J Zoo. 2012;44(2):393-398.
2. Azam, A., Younas, U., Husna, A., Ullah, N., Ali, Q., & Akhter, S. Hematological studies among bovine tuberculosis suspected herds of cattle in suburb of Islamabad, Pakistan. Wayamba. J Anim Sci 2014;6:921-926.
3. Leghari, A., Kamboh, A. A., Lakho, S. A., Khand, F. M., Malhi, K. K., Chandio, I. B., ... & Shah, J. M. Prevalence and risk factors associated with bovine tuberculosis in cattle in Hyderabad and Tando Allahyar districts, Sindh, Pakistan. Pakistan J Zool. 2020;52(1):207-212. [doi.org/10.17582/journal.pjz/2020.52.1.207.212](https://dx.doi.org/10.17582/journal.pjz/2020.52.1.207.212)
4. Khan, I. A., & Khan, A. Prevalence and risk factors of bovine tuberculosis in Nili Ravi buffaloes in the Punjab, Pakistan. Ital J Anim Sci. 2007;6(2):817-820. [doi.org/10.4081/ijas.2007.s2.817](https://doi.org/10.4081/ijas.2007.s2.817)
5. Memon, M. R., Bhutto, A. L., Khatri, P., Shah, M. G., & Memon, M. I. Prevalence and risk factor analysis of bovine tuberculosis in bovine population in Karachi. Pakistan. J Anim Health Prod. 2017;5(2):44-49. doi.org/10.17582/journal.jahp/2017/5.2.44.49
6. Ehtisham-ul-Haque, S., Javed, M. T., Ahmad, M. Z., Ahmed, I., Rafique, M. K., Irshad, I., ... & Dilbar, G. H. Monitoring the Health Status and Herd-Level Risk Factors of Tuberculosis in Water Buffalo (Bubalus bubalis) Dairy Farms in Pakistan. Pak Vet J. 2021;41(4):552-556. doi.org/10.29261/pakvetj/2021.051.
7. Javed, M. T., Farooqi, A. F., & Ullah, H. Epidemiological basis of bovine tuberculosis in buffaloes. Pak J Zool. 2009;9:417-420.
8. Arshad, M., Ifrahim, M., Ashraf, M., Rehman, S. U., & Khan, H. A. Epidemiological studies on tuberculosis in buffalo population in villages around Faisalabad. JAPS. 2012;22(3):246-249.
9. Malhi, K. K., Kamboh, A. A., Dewani, P., Kumar, C., Abro, S. H., Leghari, A., & Shoaib, M. Prevalence of bovine tuberculosis in buffaloes in Hyderabad and Tando Allahyar districts of Sindh province, Pakistan. *Buffalo Bulletin*. 2018;37(4):545-558.
10. Zahoor, M. Y. A cross-sectional study of bovine tuberculosis and its associated zoonotic risk factors in district Bahawalnagar, Punjab, Pakistan. Board of Reviewing Editors. 2021;51:192-193.
11. Javed, M. T., Shahid, A. L., Farooqi, F. A., Akhtar, M., Cardenas, G. A., Wasiq, M., & Cagiola, M. Risk factors associated with the presence of positive reactions in the SCCIT test in water buffalo around two cities in Punjab, Pakistan. Acta Trop. 2010;115(3):242-247. [doi.org/10.1016/j.actatropica.2010.04.004](https://doi.org/10.1016/j.actatropica.2010.04.004)
12. Mumtaz, N., Chaudhry, Z. I., Mahmood, N., & Shakoori, A. R. Reliability of PCR for detection of bovine tuberculosis in Pakistan. *Pak J Zool*. 2008;40(5):347-351.
13. Jalil, H. Bovine tuberculosis in Dairy Animals at Lahore, Threat to the Public Health. Metropolitan Corporation Lahore, Pakistan. 2003;11:1-11.
14. Khan, A., Chaudhry, Z. I., Shakoori, A. R., Mahmood, N., Ijaz, M., Khan, M. Z. U., ... & Ali, M. M. Detection of Mycobacterium bovis in buffaloes blood through polymerase chain reaction (PCR) and tuberculin test. JAPS. 2012;22(3):237-241.


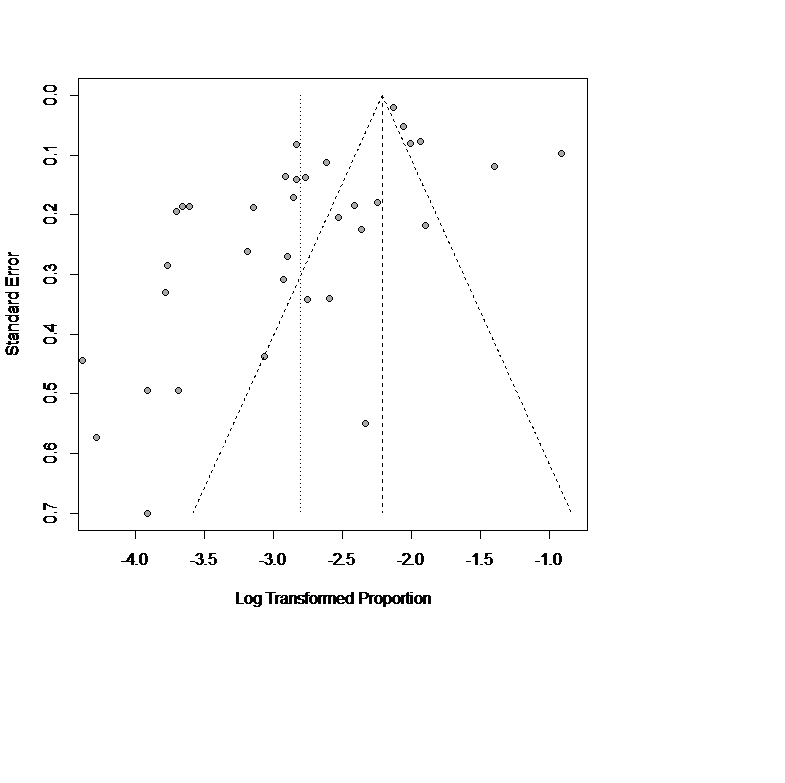


**Fig. S1** Funnel plot with pseudo 95% confidence limit intervals for the examination of publication bias in the region subgroup.


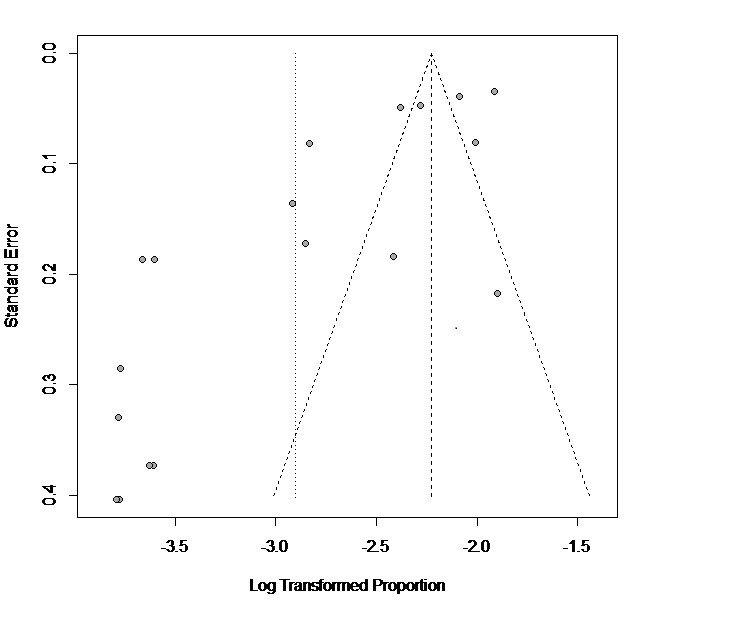


**Fig. S2**Funnel plot with pseudo 95% confidence limit intervals for the examination of publication bias in the study period subgroup.


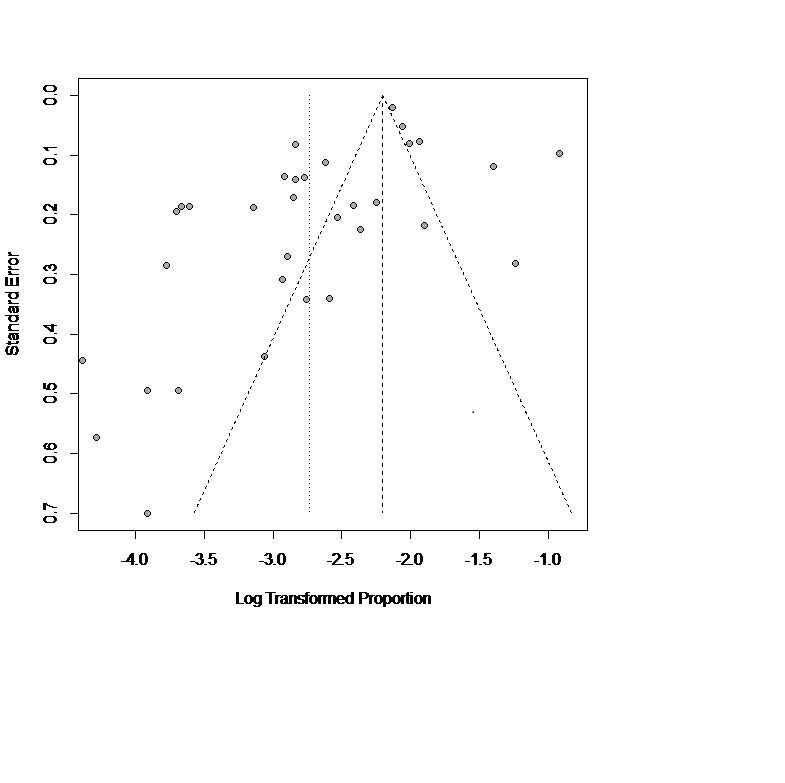


**Fig. S3** Funnel plot with pseudo 95% confidence limit intervals for the examination of publication bias in the survey area subgroup.


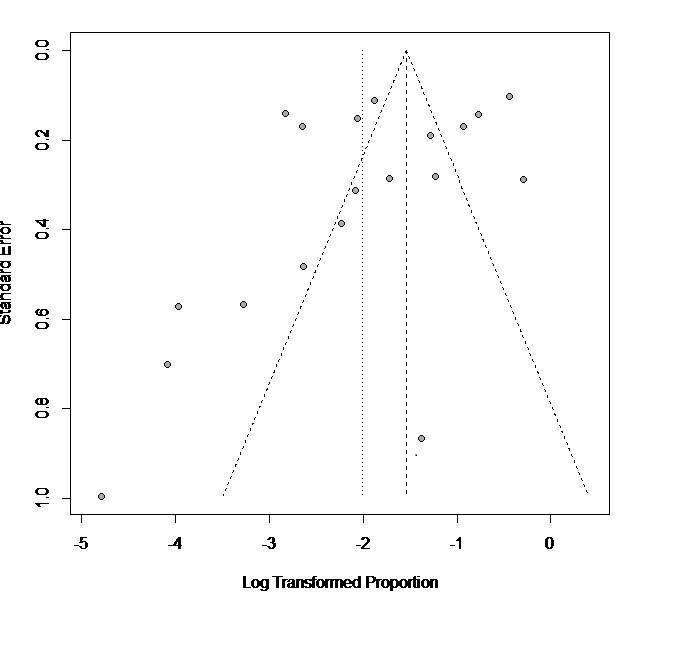


**Figure. S4** Funnel plot with pseudo 95% confidence limit intervals for the examination of publication bias in the sample classification subgroup.


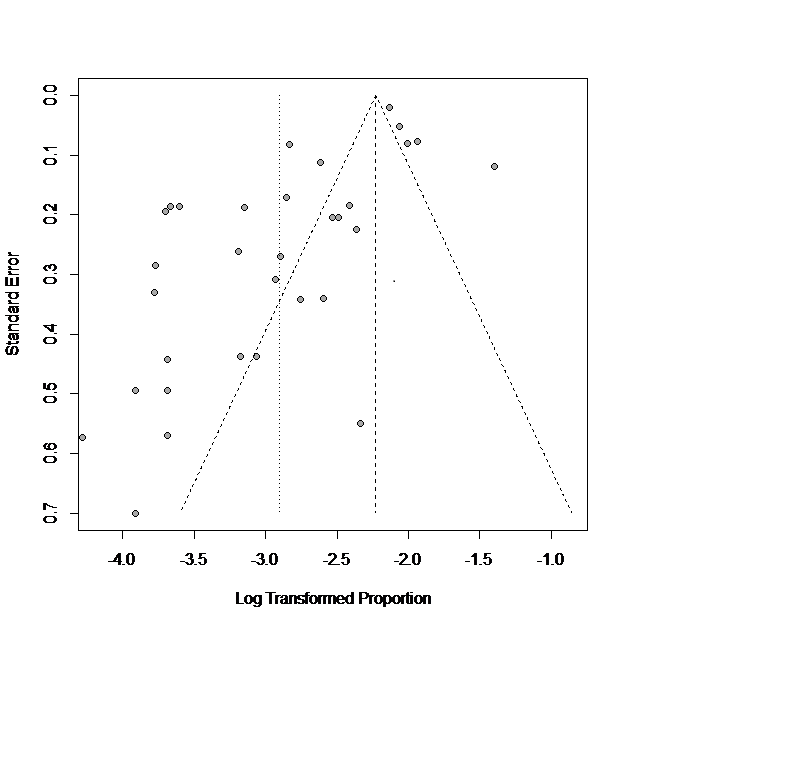


**Fig. S5** Funnel plot with pseudo 95% confidence limit intervals for the examination of publication bias in the detection method subgroup.


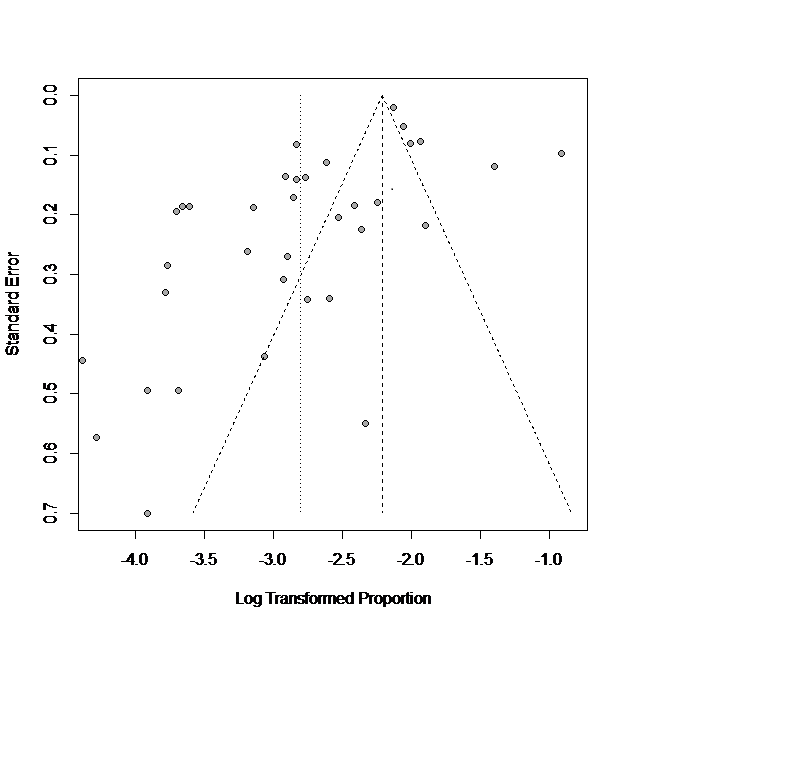


**Fig. S6** Funnel plot with pseudo 95% confidence limit intervals for the examination of publication bias in the specie subgroup.


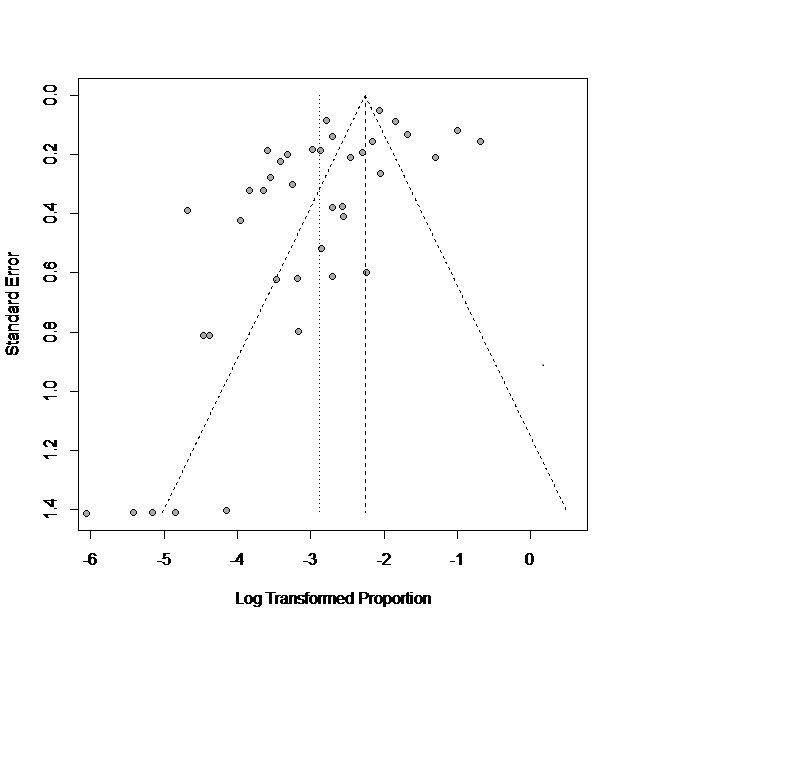


**Fig. S7** Funnel plot with pseudo 95% confidence limit intervals for the examination of publication bias in the gender subgroup.


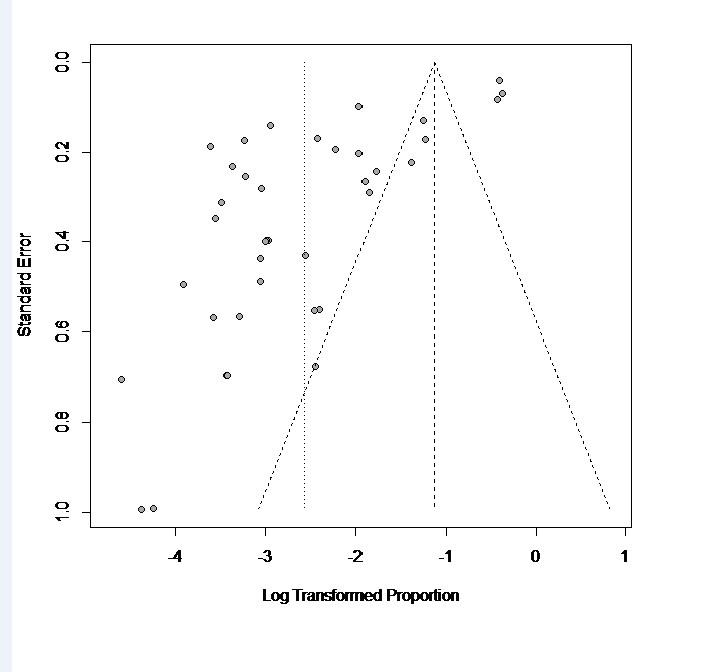


**Fig. S8** Funnel plot with pseudo 95% confidence limit intervals for the examination of publication bias in the age subgroup.


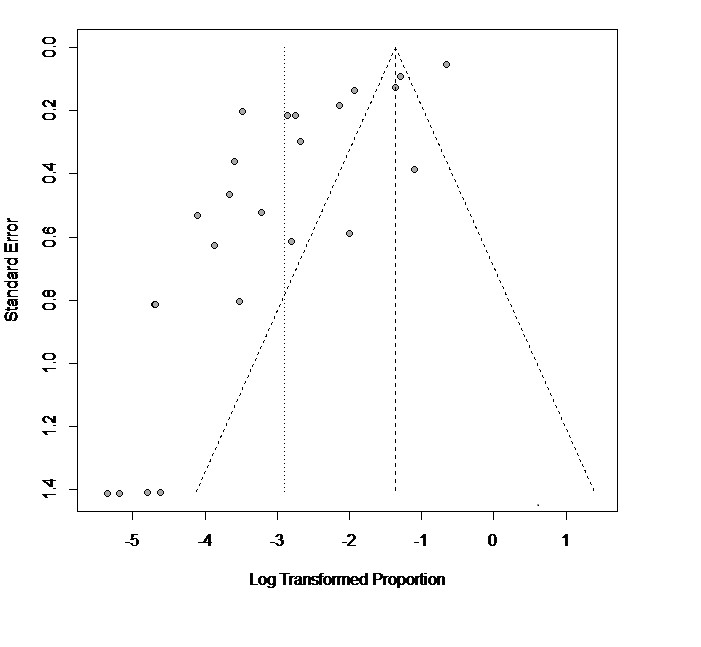


**Fig. S9** Funnel plot with pseudo 95% confidence limit intervals for the examination of publication bias in the weight subgroup.


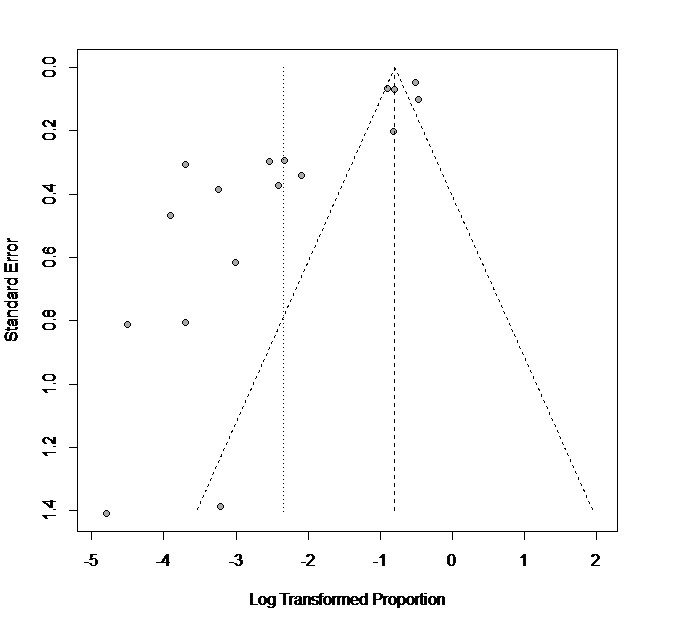


**Fig. S10** Funnel plot with pseudo 95% confidence limit intervals for the examination of publication bias in the animal status subgroup.


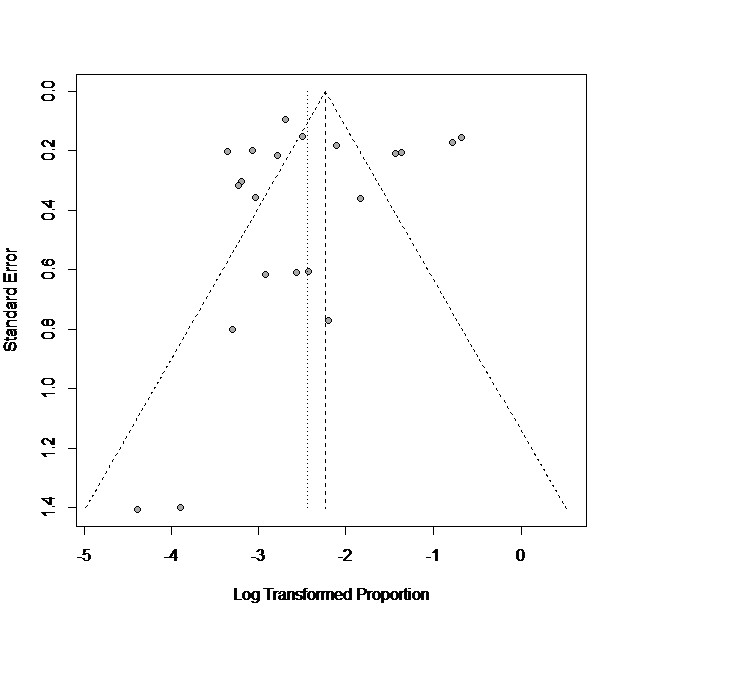


**Fig. S11** Funnel plot with pseudo 95% confidence limit intervals for the examination of publication bias in the lactation status subgroup.


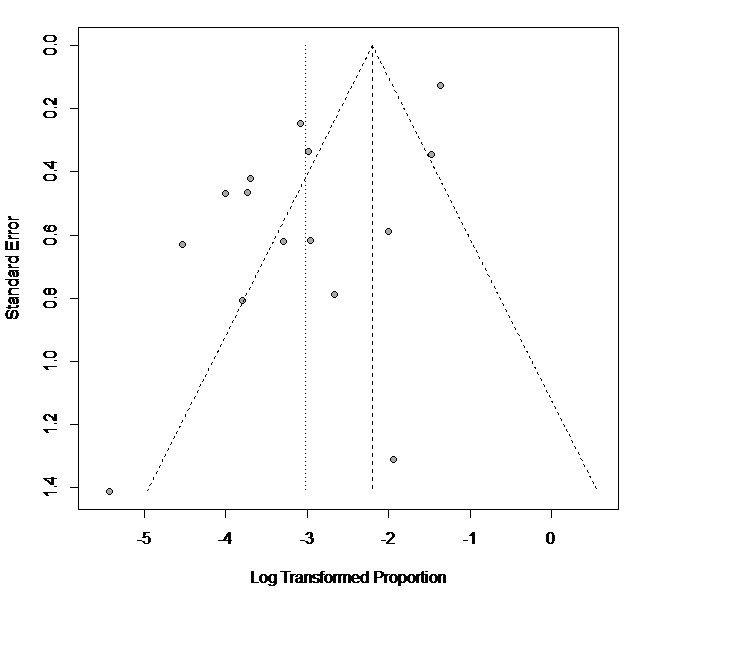


**Fig. S12** Funnel plot with pseudo 95% confidence limit intervals for the examination of publication bias in the lactation length subgroup.


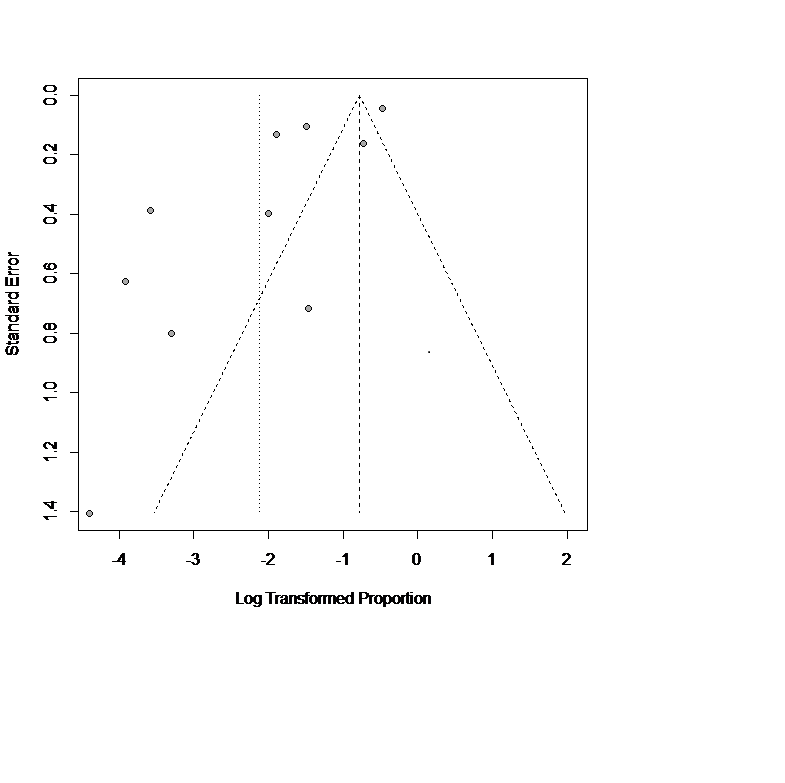


**Fig. S13** Funnel plot with pseudo 95% confidence limit intervals for the examination of publication bias in the parity subgroup.


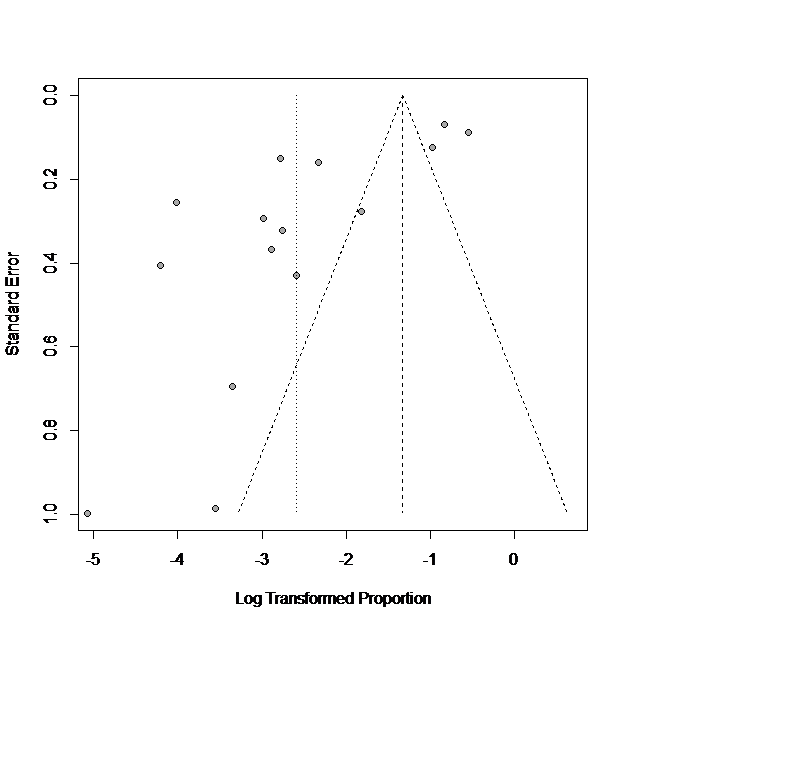


**Fig. S14** Funnel plot with pseudo 95% confidence limit intervals for the examination of publication bias in the BCS subgroup.


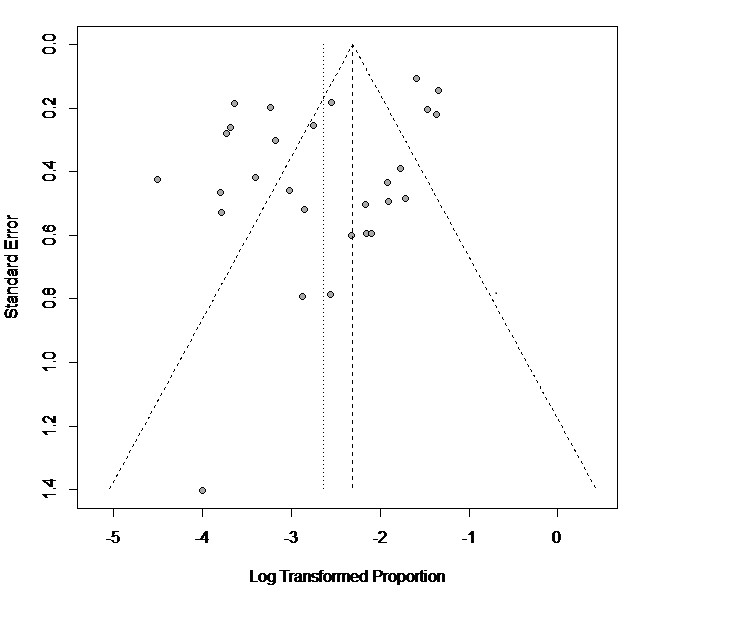


**Fig. S15** Funnel plot with pseudo 95% confidence limit intervals for the examination of publication bias in the herd size subgroup.


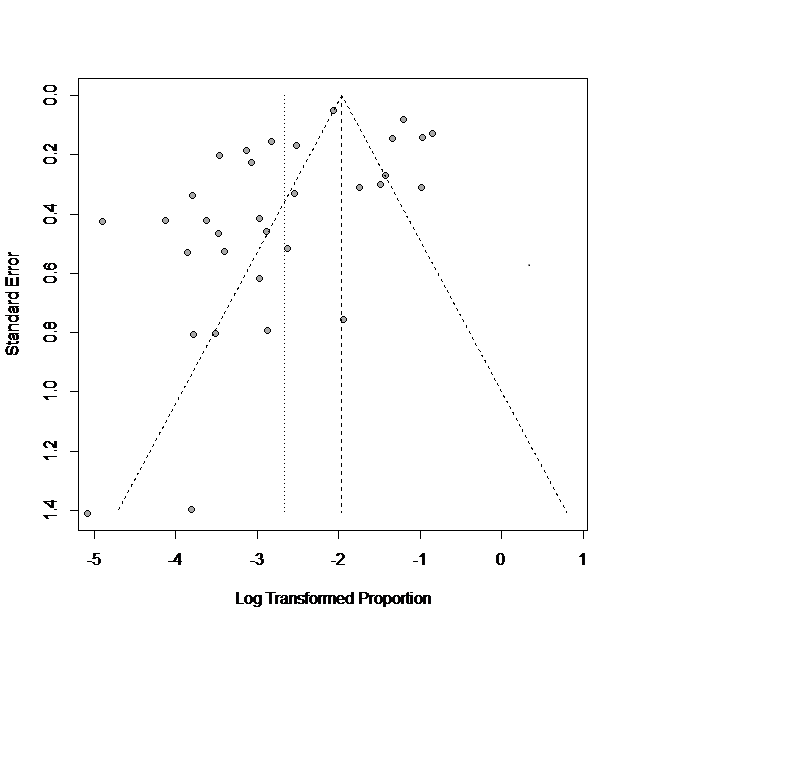


**Fig. S16** Funnel plot with pseudo 95% confidence limit intervals for the examination of publication bias in the breed subgroup.


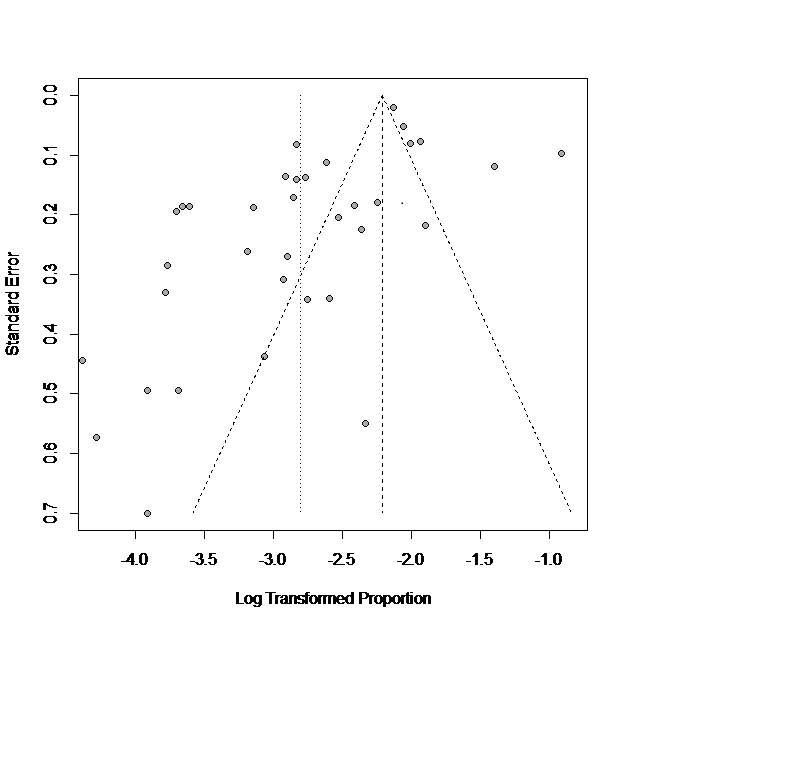


**Fig. S17** Funnel plot with pseudo 95% confidence limit intervals for the examination of publication bias in the quality level subgroup.


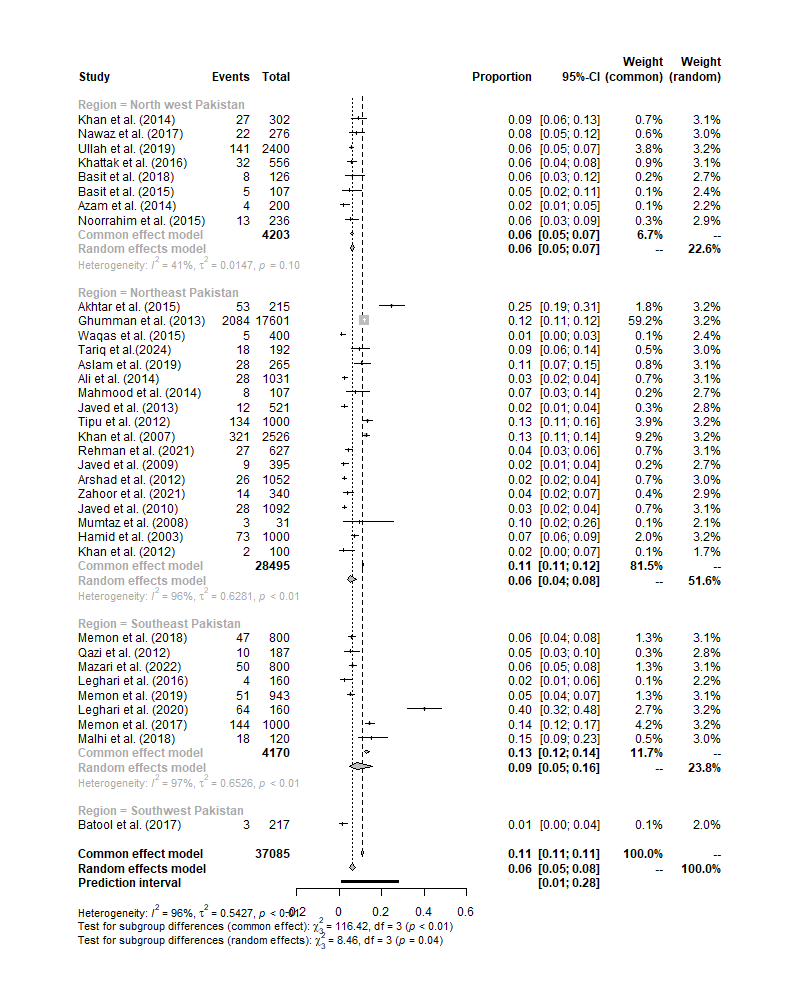


**Fig. S18** Forest plot of the region subgroup.


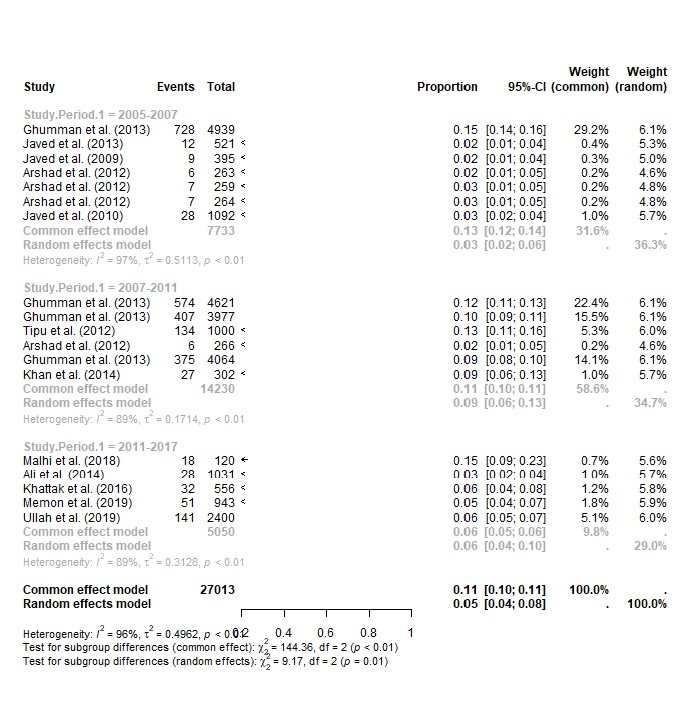


**Fig. S19** Forest plot of the study period subgroup.


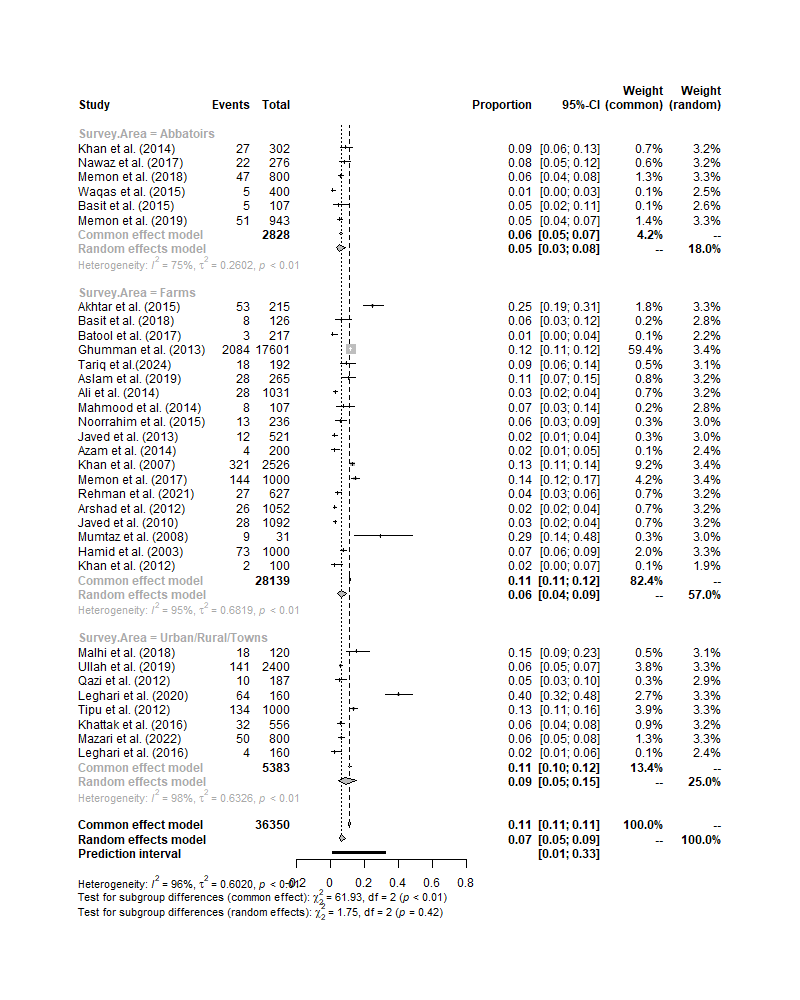


**Fig. S20** Forest plot of the survey area subgroup.


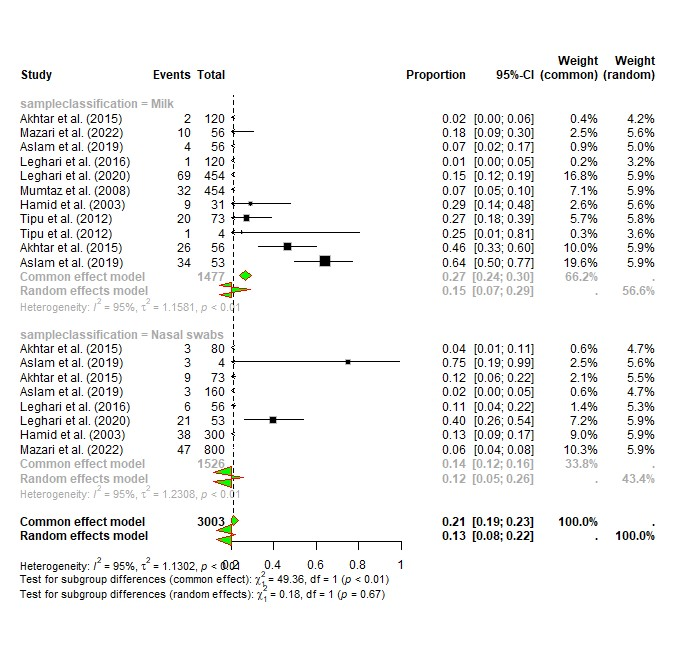


**Fig. S21** Forest plot of the sample classification subgroup.


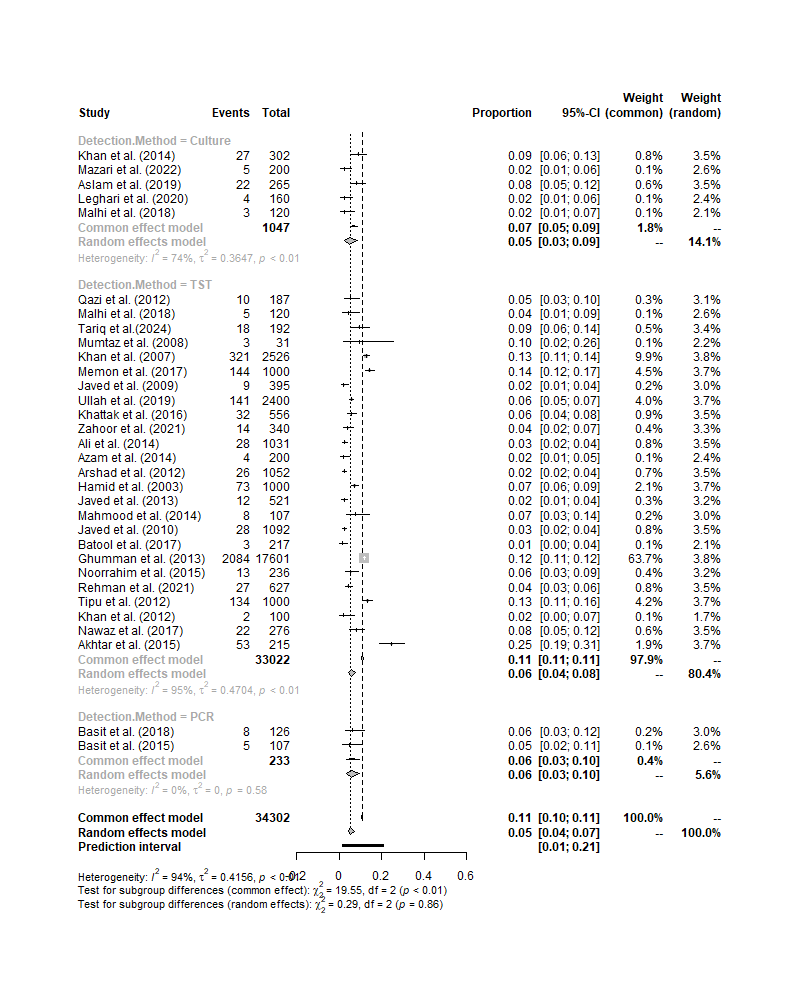


**Fig. S22** Forest plot of the detection method subgroup.


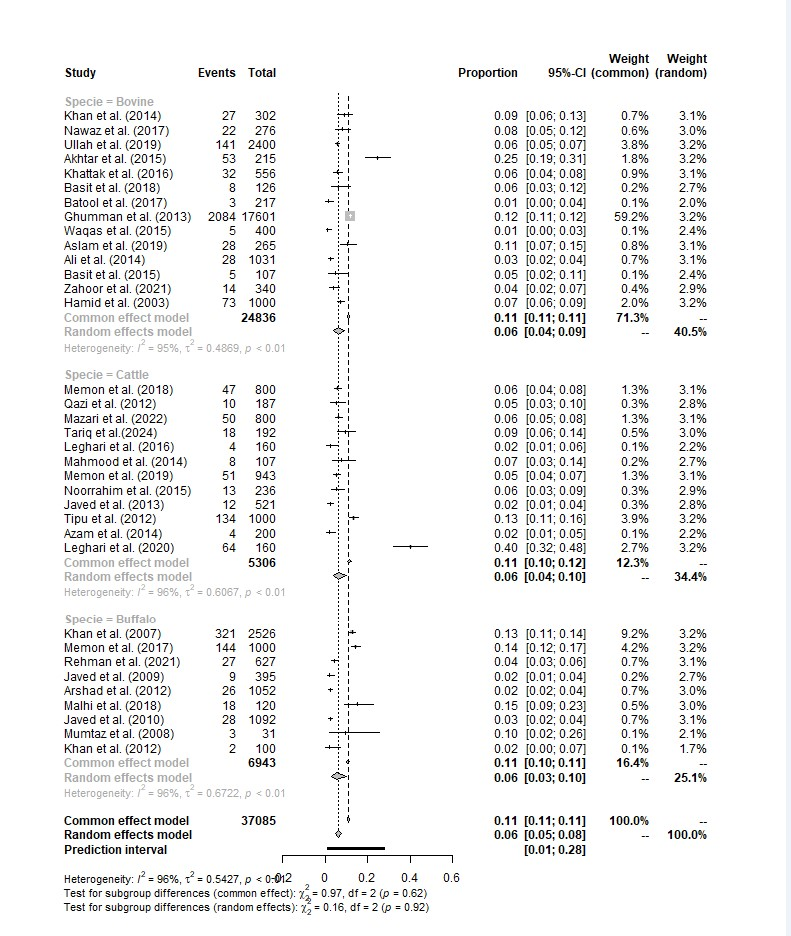


**Fig. S23** Forest plot with of the specie subgroup.


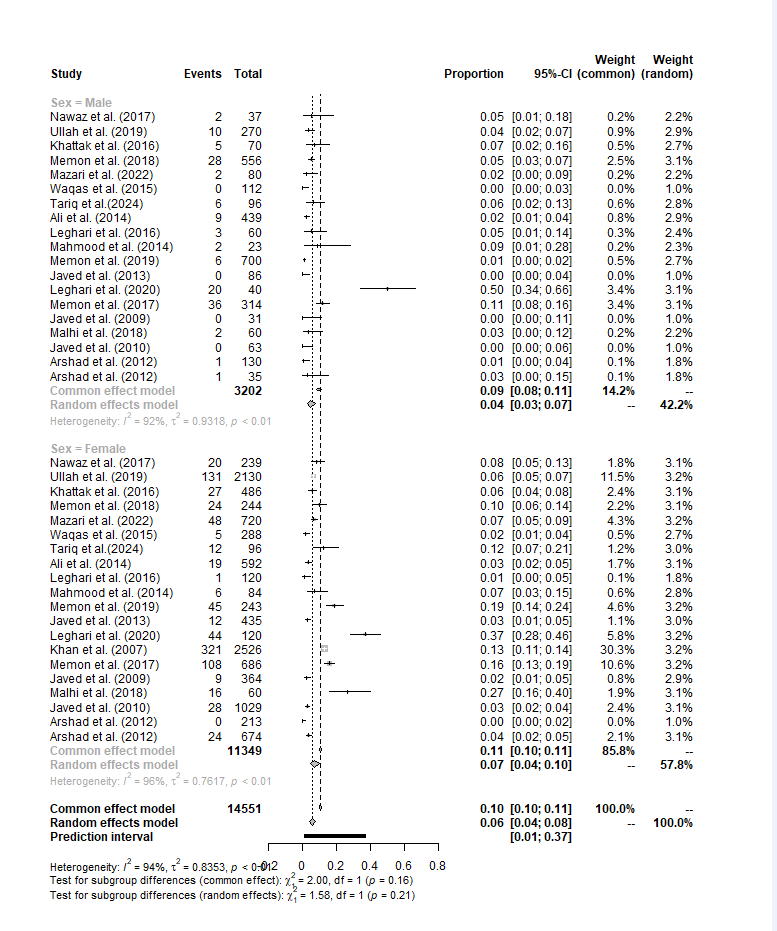


**Fig. S24** Forest plot of the gender subgroup.


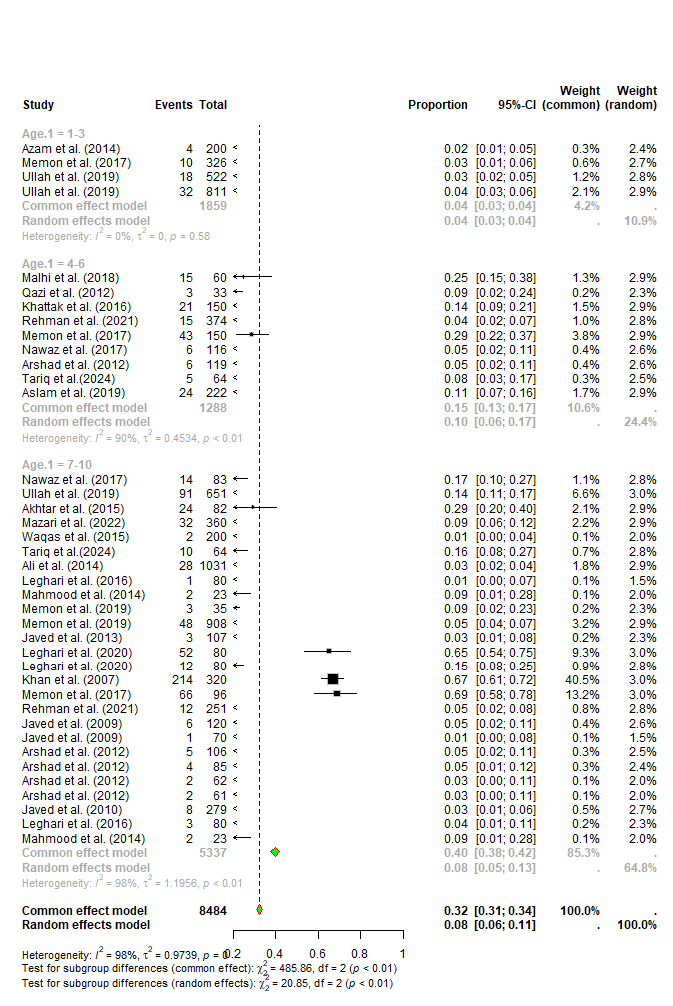


**Fig. S25** Forest plot of the age subgroup.


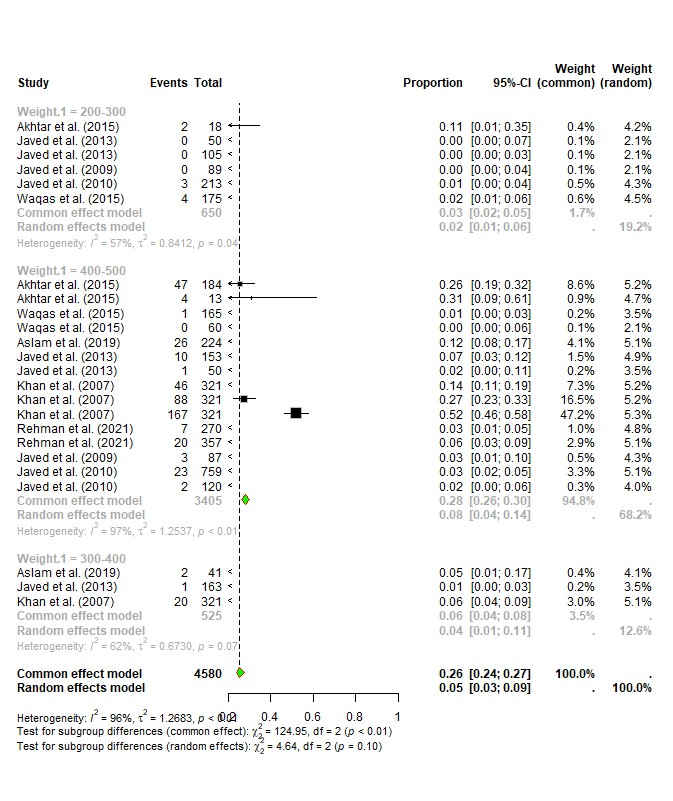


**Fig. S26** Forest plot of the weight subgroup.


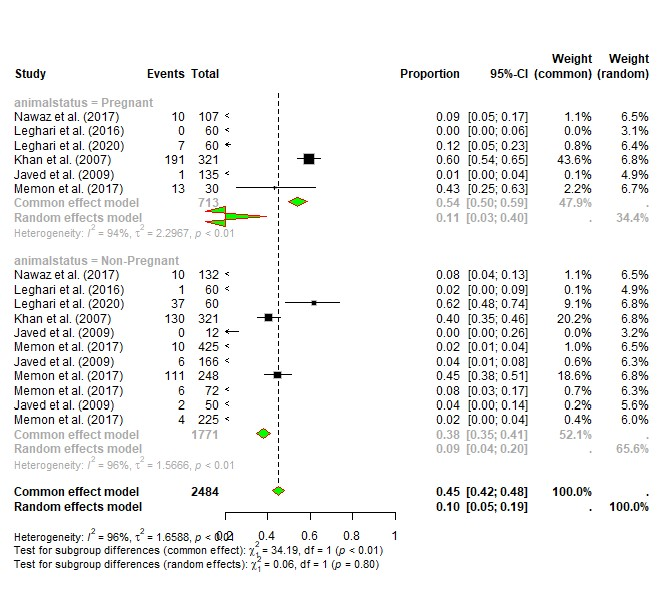


**Fig. S27** Forest plot of the animal status subgroup.


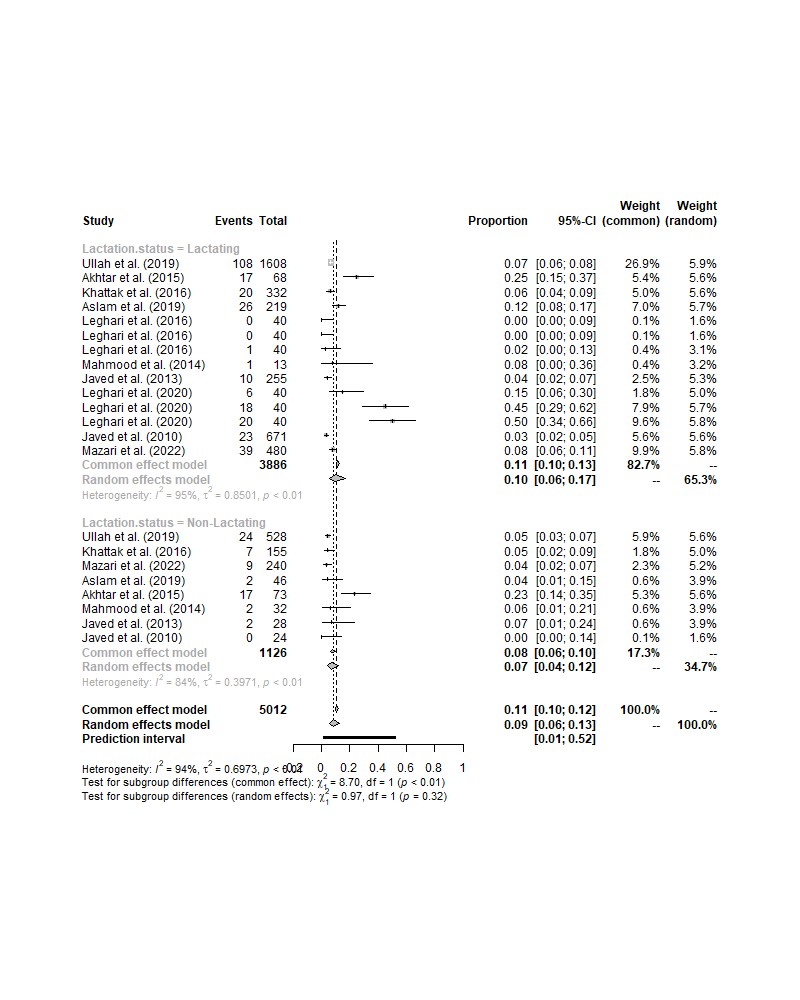


**Fig. S28** Forest plot of the lactation status subgroup.


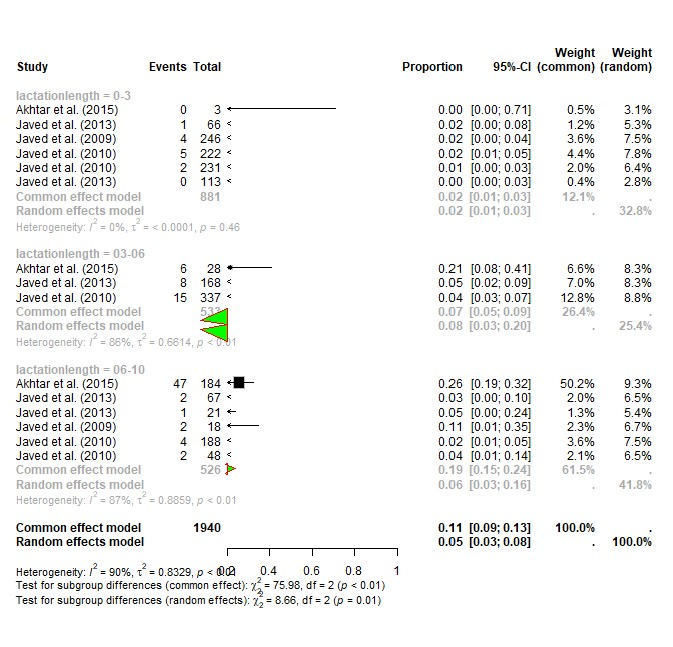


**Fig. S29** Forest plot of the lactation length subgroup.


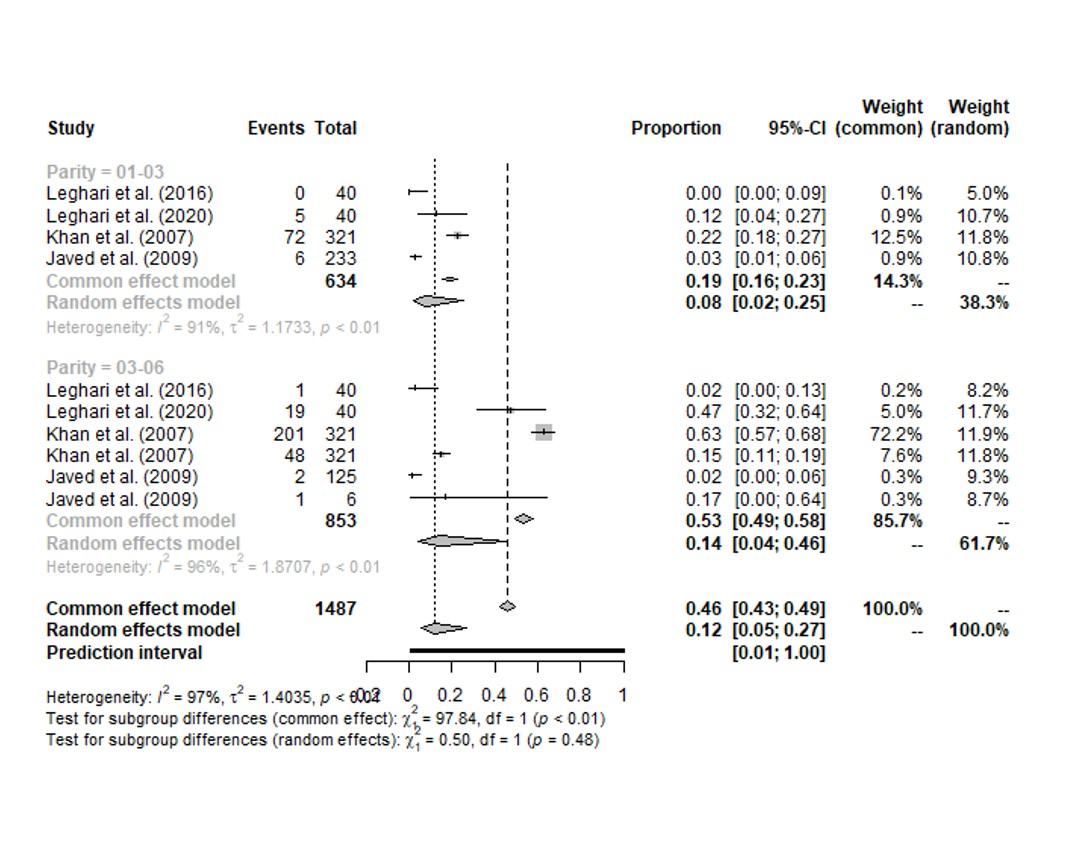


**Fig. S30** Forest plot of the parity subgroup.


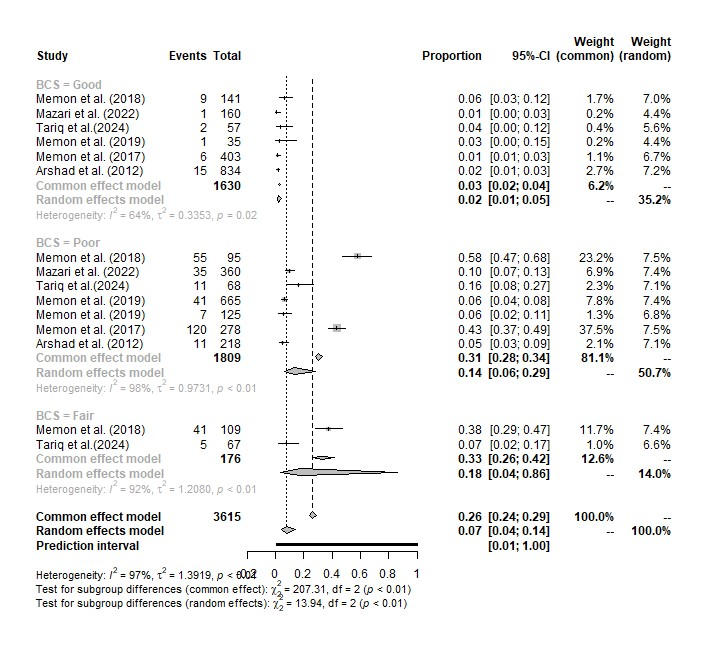


**Fig. S31** Forest plot of the BCS subgroup.


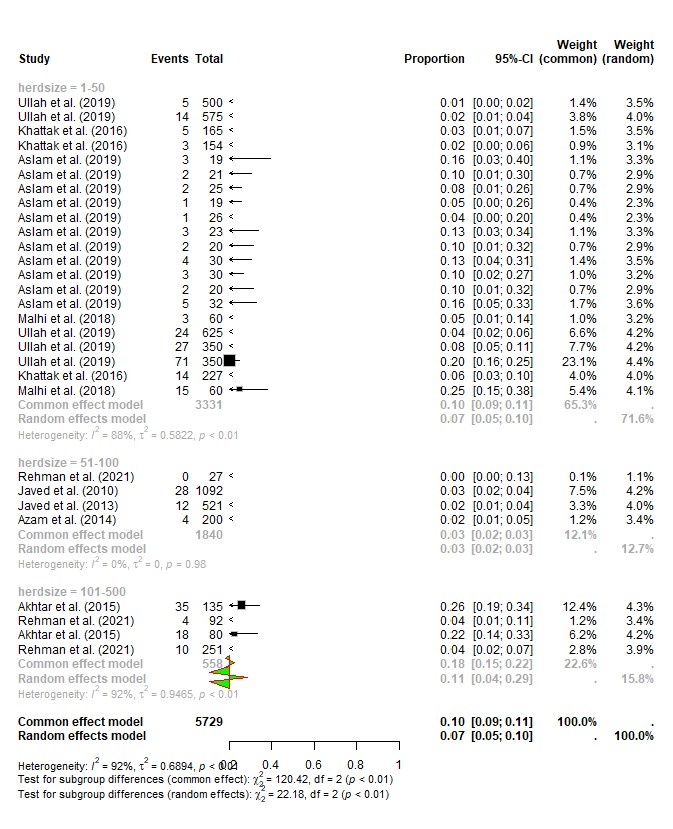


**Fig. S32** Forest plot of the herd size subgroup.


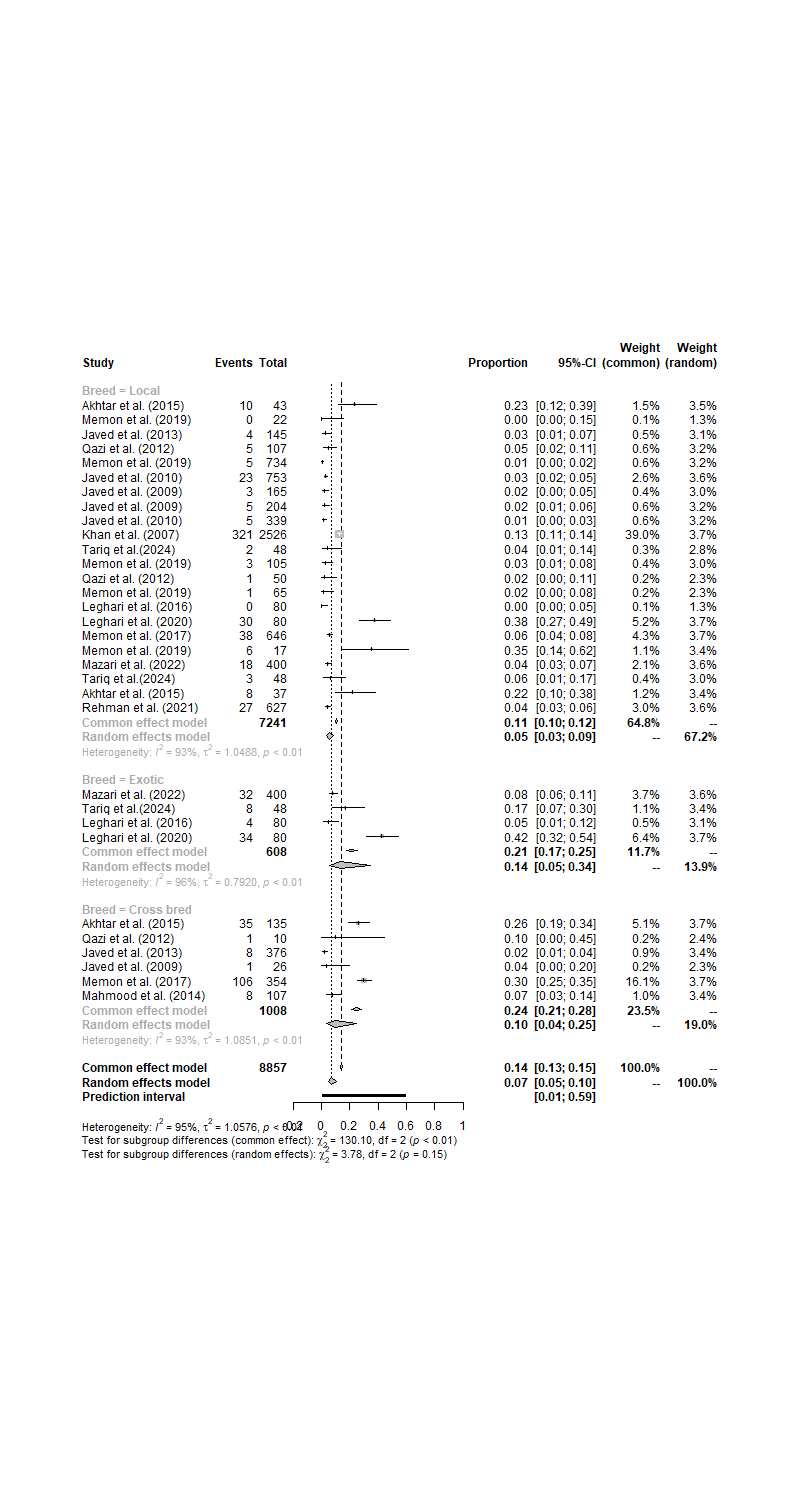


**Fig. S33** Forest plot of the breed subgroup.


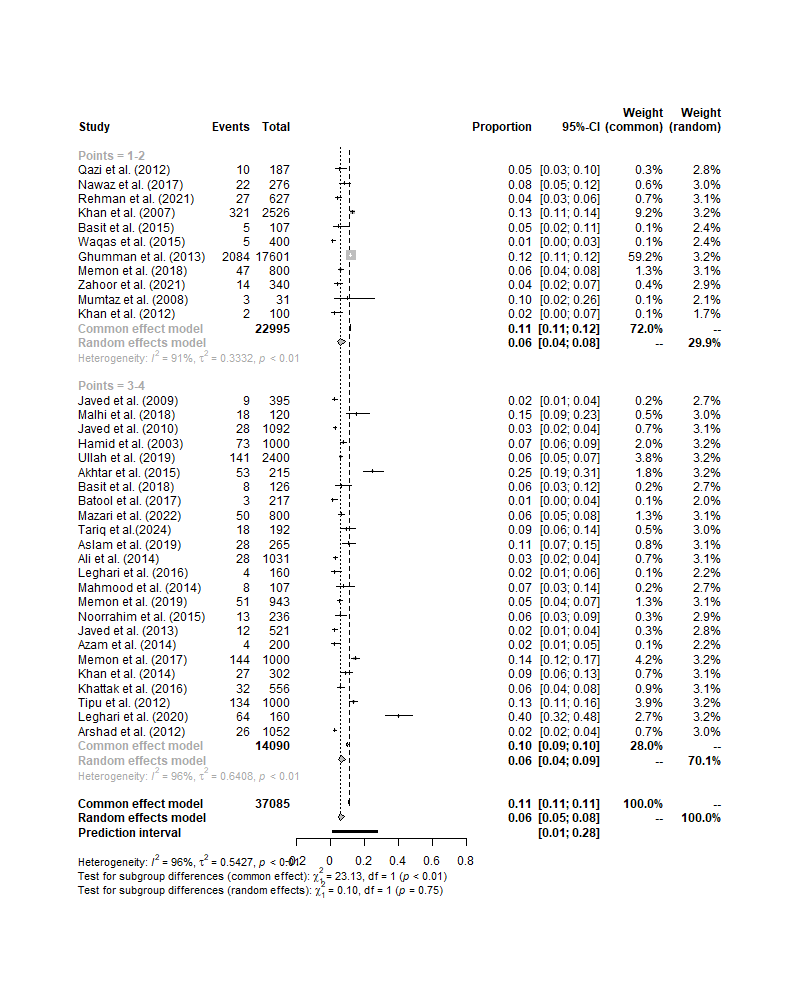


**Fig. S34** Forest plot of the quality level subgroup.
